# Parental violent offending and offspring suicidal behavior: a nationwide register-based study

**DOI:** 10.1017/S0033291726103717

**Published:** 2026-04-01

**Authors:** Aurora Järvinen, Seena Fazel, Ralf Kuja-Halkola, Isabell Brikell, Zheng Chang, Brian M. D’Onofrio, Henrik Larsson, Paul Lichtenstein, Antti Latvala

**Affiliations:** 1 Institute of Criminology and Legal Policy, University of Helsinki, Helsinki, Finland; 2 Department of Medical Epidemiology and Biostatistics, Karolinska Institutet, Stockholm, Sweden; 3 Department of Psychiatry, University of Oxford, Oxford, UK; 4 Department of Global Public Health and Primary Care, University of Bergen, Bergen, Norway; 5 Department of Biomedicine, Aarhus University, Aarhus, Denmark; 6 Department of Psychological and Brain Sciences, Indiana University Bloomington, Bloomington, USA; 7 School of Medical Sciences, Örebro University, Örebro, Sweden

**Keywords:** family factors, intergenerational effects, parental offending, population-based, risk stratification, suicidal behavior, violent crime

## Abstract

**Background:**

Parental violent offending and offspring suicidal behavior are associated, but a deeper understanding of the risk within this population is needed to best identify and support those most in need. This study examined the risk of suicidal behavior among offspring of parents with violent convictions, primarily aiming to identify high-risk subgroups.

**Methods:**

The study included 2,956,465 individuals born in Sweden 1977–2010, and their parents. Data were obtained from nationwide registers available until the end of 2020. The authors examined the risk of suicidal behavior among offspring with none, one, or both parents with violent convictions by offspring’s age 10, and further investigated the risk among exposed offspring by parental psychiatric disorders, child–parent coresiding, and other factors. Children-of-siblings analyses were conducted to better understand the nature of the association.

**Results:**

There were 254,793 (8.6%) and 11,777 (0.4%) offspring with one or both parents with violent convictions. Absolute risk of suicidal behavior was highest among those with both parents convicted; 14.3% (95% CI, 13.0–15.7) of male and 16.6% (95% CI, 15.3–18.0) of female offspring engaged in suicidal behavior by age 30, compared to 4%–4.5% of offspring of parents without convictions. The more adversities accumulated in families with parental offending, the higher the cumulative incidence of suicidal behavior. Genetic factors partly accounted for the association.

**Conclusions:**

Offspring of parents with violent convictions are a group at high risk of suicidal behavior in need of early identification, multiagency coordination, and measures to reduce the risk of self-harm and suicide.

## Introduction

Offspring of parents who engage in criminal behavior are a high-risk group for mental disorders (Luk et al., 2023; Wildeman, Goldman, & Turney, 2018), with parental violent offending linked to a particularly elevated risk (Järvinen et al., 2024). Less is known about the risk of self-harm in this population, despite the close link between self-harm and mental health problems (McEvoy et al., 2023). To date, only a few studies have specifically investigated the link between parental criminality and offspring self-harm (Bravo et al., 2024; Cumming et al., 2023; Davis & Shlafer, 2017; O’Hare et al., 2022). These studies have used varying definitions of self-harm, with some focusing solely on self-injurious behaviors (Cumming et al., 2023), while others expand the definition to include suicidal ideation (e.g. Bravo et al., 2024). Despite these differences, a link between parental offending and offspring self-harm has generally been found. However, a more nuanced understanding of the risk of self-harm within this group is still needed.

Merely identifying the association between parental criminality and offspring self-harm is not sufficiently informative for guiding policy makers and public health services, who often face the challenge of allocating limited resources to those most in need. Clarifying the risk of self-harm in greater detail among offspring of parents with criminal offending can yield clinically relevant and actionable insights. A better understanding of how the risk of self-harm in this population varies according to other parental characteristics and adversities commonly occurring in these families allows for the identification of particularly vulnerable subgroups. For example, previous studies have often failed to distinguish specific characteristics of exposure to parental criminality, such as maternal versus paternal offending (Bravo et al., 2024; O’Hare et al., 2022). Further, psychiatric disorders are common among people with criminal offending (Gottfried & Christopher, 2017; Whiting, Lichtenstein, & Fazel, 2021), and there is a strong association between parental psychiatric disorders and offspring self-harm (Mok et al., 2016; O’Hare et al., 2022). Self-harming behavior may thus be particularly common among offspring exposed to both parental offending and parental psychiatric disorders. Moreover, not only parental offending but also parental incarceration has been associated with offspring self-harm (Cumming et al., 2023; Davis & Shlafer, 2017; O’Hare et al., 2022), but it is unknown whether exposure to parental incarceration is associated with a further increase in risk of self-harm among offspring of parents with criminal offending.

Importantly, besides possible subgroup differences in risk for self-harm, it remains unclear to what extent the association between parental crime and offspring self-harm is explained by shared familial factors rather than parental offending itself. Families with parental offending are a selected group in many respects, and adjusting for measured covariates is likely insufficient for ruling out selection effects. Both violent offending and self-harm are partly explained by genetic factors (Frisell, Pawitan, Långström, & Lichtenstein, 2012; Pedersen & Fiske, 2010). Crucially, violence and self-harm are robustly associated, known as dual-harm behavior (O’Donnell, House, & Waterman, 2015; Richmond-Rakerd et al., 2019; Sahlin et al., 2017), and this co-occurrence is likely partly due to shared genetic background (Brent & Melhem, 2008; Mann et al., 2009). It is thus important to consider the overlapping genetic etiology of these behaviors for a better understanding of the possible impact of parental violent crime on offspring self-harm. A clearer understanding of the extent to which the link may be causal can inform the design of best practices to support this at-risk group.

We conducted a longitudinal population-based study on the risk of suicidal behavior among offspring of parents with violent offending. Suicidal behavior was defined as nonfatal self-harming events diagnosed in specialized healthcare and deaths by suicide, thus representing the most severe forms of self-harm. We had three aims. First, we described the risk of suicidal behavior among offspring with none, one, or both parents convicted of violent offenses. Second, aiming to identify possible high-risk subgroups, we investigated how the risk of suicidal behavior among offspring varied by further exposure to parental psychiatric disorders, parental incarceration, and other factors, examining maternal and paternal offending separately. Finally, to better understand the mechanisms driving the association, we compared the risk between differentially exposed children of siblings to partly account for genetic influences underlying the association.

## Methods

Data were obtained from several Swedish nationwide registers, maintained by government agencies, and linked via the personal identification number assigned to all citizens at birth and for immigrants upon arrival in Sweden. At the time of the study, register data were available until the end of 2020. As all data have been anonymized for research purposes, personal consent for the use of registers was not required. The Regional Ethical Review Board of Stockholm has approved the use of register linkages (Dnr 2020–06540). Registers used in the study are listed in Supplementary Table S1.

### Study population

The study population included individuals born in Sweden between January 1, 1977, and December 31, 2010 (*N* = 3,505,960), identified from the Total Population Register of Statistics Sweden (Ludvigsson et al., 2016). The cohort was selected to maximize sample size while ensuring all offspring had full data on suicidal behavior from age 10 years onwards, as suicidal behavior-related diagnoses were relatively uncommon before 1987. We used the Multi-Generation Register to identify biological parents of each individual, excluding children with missing information on either parent (*N* = 47,072) (Ekbom, 2011). Aiming to minimize missing data in the exposure, for which information was available from 1973 onwards, we further restricted the sample to individuals with parents born after December 31, 1949, resulting in a final study sample of 2,956,465 individuals and their parents (*N* = 2,983,640).

In analyses stratified by the child’s and parent’s coresiding, we restricted the study population to offspring born between January 1, 1982, and December 31, 2010, as residential data were available from 1982 onwards and coresiding during the first 10 years of the child’s life was examined. This resulted in a sample of 2,752,991 individuals and their parents (*N* = 2,782,498).

For children-of-siblings analyses, we further linked the parent generation to their parents (i.e. grandparents of the offspring) to determine different sibling types (twins, full siblings, and paternal/maternal half-siblings) in the parent generation, including only those with information on both parents (for details, see Appendix S1 of the Supplementary Material).

### Measures

#### Exposure

We used three different definitions for parental violent offending, as detailed in Appendix S1 of the Supplementary Material and Supplementary Table S2. In the main analyses, we defined parental violent offending as criminal convictions for violent or sexual offenses of none, one, or two parents, obtained from the National Crime Register (Brottsförebyggande rådet, 2024). We further created a variable indicating the number of parental violent convictions during the first 10 years of the child’s life. In stratified analyses and children-of-siblings analyses, we defined the exposure as a dichotomous variable and studied paternal and maternal convictions separately. For all exposures, we included parental convictions before the child’s 10th birthday to ensure a correct temporal ordering of exposure and outcome.

#### Outcome

We defined offspring suicidal behavior as having a diagnosis of nonfatal intentional self-harm or an event of undetermined intent, or death by suicide or from undetermined intent. Both nonfatal events and deaths were included in the main outcome, consistent with previous research (O’Reilly et al., 2020), but we also conducted main analyses and stratified analyses separately for nonfatal events and deaths. Events of undetermined intent were included to ensure that information on misclassified events of self-harm was also obtained, a practice supported by previous epidemiological studies (Bakst et al., 2016; Edwards et al., 2021; Simon et al., 2018). However, we also conducted a sensitivity analysis excluding events and deaths of undetermined intent. We included diagnoses and deaths from age 10 up to a maximum of age 30, using information on the first event in the analyses.

Diagnostic information according to the International Classification of Diseases (ICD) was retrieved from the National Patient Register, which covers inpatient and outpatient diagnoses in specialized healthcare since 1973 and 2001, respectively (Ludvigsson et al., 2011). The Cause of Death Register provided death dates and ICD codes for causes of death (Brooke et al., 2017). The outcomes and corresponding ICD-8, ICD-9, and ICD-10 codes are described in Supplementary Table S3.

#### Stratifying variables and covariates

We conducted the main analyses stratified by offspring sex. In analyses aiming to identify possible high-risk subgroups, we used five dichotomous stratifying variables: (i) paternal/maternal psychiatric disorders, defined as any mental or behavioral disorder before the child’s 10^th^ birthday (from the National Patient Register, ICD codes in Supplementary Table S3), (ii) paternal/maternal externalizing disorders before the child’s 10^th^ birthday (ICD codes in Supplementary Table S3), (iii) paternal/maternal suicidal behavior before the child’s 10^th^ birthday (ICD codes in Supplementary Table S3), (iv) paternal/maternal incarceration, including all custodial sentences served in prison or other closed institutions before the child’s 10th birthday, from the National Crime Register (Brottsförebyggande rådet, 2024), and (v) the child’s and parent’s coresiding, that is whether the child had lived in the same area as his/her parent for at least 8 years of the first 10 years of life, based on the DeSO (Demographic Statistical Areas) classification (Statistics Sweden, 2024).

As covariates, we included the child’s birth year (treated as categorical), parents’ birth years (categorical), and parental immigration status (from the Total Population Register, defined as being born in Sweden or in another country). In children-of-siblings analyses, we further conducted more adjusted models also including highest parental education (from the National Census before 1990 and from the Longitudinal Integration Database for Health Insurance and Labor Market Studies from 1990 to 2020 as an ordinal variable with four categories ranging from unfinished compulsory education to a postgraduate degree), parental psychiatric disorders before the child’s 10th birthday, and co-parent’s violent convictions before the child’s 10th birthday.

### Statistical analyses

#### Aim 1: Description of risk

To address our first research aim, we estimated the cumulative incidence of suicidal behavior for offspring with none, one, or two parents convicted of violent offenses. We used Kaplan–Meier survival estimates under the assumption of no competing risks, with age as the underlying timescale. Individuals were followed up from age 10 until the date of the first suicidal event, emigration (data from the Migration Register), death, age 30, or the end of follow-up (31 December 2020), whichever occurred first. We further fitted Cox proportional hazard regression models to estimate population-level associations between parental violent convictions and offspring suicidal behavior. The models provided hazard ratios (HRs) estimating the relative hazard of suicidal behavior during the follow-up among those with one or two parents with convictions as compared to those without convicted parents, adjusting for covariates. The assumption of proportional hazards was evaluated by visual inspection of Schoenfeld residuals. To describe the risk in more detail, we estimated the cumulative incidence of suicidal behavior according to the number of paternal/maternal violent convictions and examined the association between the number of paternal/maternal violent convictions and the risk of offspring suicidal behavior.

#### Aim 2: Identification of high-risk subgroups

For our second research aim, we conducted stratified analyses estimating the cumulative incidence of suicidal behavior for individuals with and without convicted fathers/mothers, stratified by (i) paternal/maternal psychiatric disorders, (ii) paternal/maternal externalizing disorders, (iii) paternal/maternal suicidal behavior, (iv) paternal/maternal incarceration, and (v) the child’s and father’s/mother’s coresiding. For each analysis, we created a four-level exposure variable with mutually exclusive categories, for example, (1) no paternal violent convictions or paternal psychiatric disorders; (2) no paternal violent convictions but paternal psychiatric disorders; (3) paternal violent convictions but no paternal psychiatric disorders; and (4) paternal violent convictions and paternal psychiatric disorders. We tested differences in absolute risks of offspring suicidal behavior between paternal and maternal convictions. *P*-values were obtained by performing a Wald-test of the difference in cumulative incidence between exposure groups. Standard errors for these tests were found through the standard deviations of differences of estimates from 1,000 clustered bootstrapping repeats, where clustered resampling was on family identifiers. This handles dependencies between paternal and maternal estimates (since each individual contributes to both estimates), as well as dependencies between siblings in families (since they share parents).

To assess the relative contributions of the above-mentioned factors to the association between parental violent offending and offspring suicidal behavior, we conducted separate Cox regression models, each stratified by one of these factors. To further examine each factor’s unique association with offspring suicidal behavior while holding other variables constant, we performed multivariable Cox regression analyses including parental violent convictions along with the other factors as predictors in the model, conducting separate models for paternal and maternal factors.

To examine the risk of suicidal behavior in relation to parental violent convictions and the accumulation of the other parental factors described above, we estimated the cumulative incidence of suicidal behavior for offspring unexposed to paternal/maternal violent convictions and other parental factors versus offspring exposed to paternal/maternal violent convictions, further dividing the latter group into subgroups based on the number of other parental factors present (ranging from 0 to 4). We also conducted Cox regression analyses to estimate the risk of suicidal behavior in these exposure groups as compared to offspring not exposed to parental violent convictions or other factors.

#### Aim 3: Examination of underlying mechanisms

To address our final research aim, we performed children-of-siblings analyses (D’Onofrio et al., 2003, 2020; McAdams et al., 2014). Using information on siblings from the parental generation, we first identified three types of children of siblings (i.e. cousins) in the offspring generation with different levels of genetic relatedness: children of half-siblings, full siblings/dizygotic (DZ) twins, and monozygotic (MZ) twins. We fitted separate stratified Cox regression models within these three clusters of children of siblings (for details, see Appendix S1 of the Supplementary Material). These models estimate a different baseline hazard for each cluster (i.e. stratum) and provide estimates for how differences between children of siblings in the predictor variable affect their deviance from their shared baseline hazard. More specifically, the models compare the risk of suicidal behavior between children of siblings who are discordant for exposure to parental violent convictions and rule out all (measured and unmeasured) factors shared between extended family members (D’Onofrio et al., 2003, 2020; McAdams et al., 2014). As unexposed children in these three types of extended family clusters share different proportions of genes with their convicted aunt/uncle, the models rule out varying levels of genetic influences on the intergenerational association: the models for children of half-siblings, full siblings/DZ twins, and MZ twins rule out 25%, 50%, and 100% of the genetic influences shared between the convicted parent and his/her child, respectively (for details, see Appendix S1 of the Supplementary Material and Supplementary Figure S1). Comparing the risk of suicidal behavior across the models with increasing control for genetic effects thus provides insight into the role of genetic factors behind the association. We tested the statistical significance of the differences between the estimates by examining the interaction term between parental violent convictions and extended family type.

#### Sensitivity analyses

We performed several sensitivity analyses. First, we conducted an analysis using a more conservative definition of the outcome, excluding events and deaths of undetermined intent. We examined both the cumulative incidence of this more narrowly defined outcome and the population-level association between parental violent offending and the outcome. Second, we studied the association between parental violent offending and offspring suicidal behavior, restricting the sample to children whose parents were born from 1958 onwards to ensure that all parents had full information on violent offending from the age of 15 (the age of criminal responsibility in Sweden), as conviction data were only available from 1973 onwards. Finally, to account for exposure to parental violent convictions over the entire follow-up period, we conducted a sensitivity analysis including all parental violent convictions before the child’s 30th birthday.

All analyses were conducted with Stata, version 18 (StataCorp, 2023).

## Results

Table 1 gives descriptive information on offspring with none, one, or both parents convicted of violent offenses. The number of offspring with one or both parents convicted was 254,793 (8.6%) and 11,777 (0.4%), respectively. Table 2 shows the cumulative incidence of suicidal behavior from age 10 to age 30 among male and female offspring with none, one, or both parents convicted. The cumulative incidence of suicidal behavior was highest among offspring whose both parents had been convicted and appeared higher for female than for male offspring. Specifically, 14.3% (95% CI, 13.0%–15.7%) of male and 16.6% (95% CI, 15.3%–18.0%) of female offspring with both parents convicted engaged in suicidal behavior by age 30. When considering the cumulative incidence of nonfatal self-harm and suicide separately, we found that both were highest for offspring with both parents convicted. However, the cumulative incidence of nonfatal self-harm appeared overall higher among females, whereas that of suicide appeared higher among males.

**Table 1.** Characteristics of offspring born between 1977 and 2010 (*N* = 2,956,465) with none (*N* = 2,689,895), one (*N* = 254,793), or both parents (*N* = 11,777) with violent convictions

	Offspring of parents without violent convictions (*N* = 2,689,895)	Offspring with father convicted of violent offenses (*N* = 235,802)	Offspring with mother convicted of violent offenses (*N* = 18,991)	Offspring with both parents convicted of violent offenses (*N* = 11,777)
Sex, *N* (%)				
Men	1,382,449 (51.4)	121,428 (51.5)	9,846 (51.9)	6,029 (51.2)
Women	1,307,446 (48.6)	114,374 (48.5)	9,145 (48.2)	5,748 (48.8)
Birth year, median (1st and 3rd quartiles)	1995 (1989, 2004)	1996 (1989, 2004)	1999 (1991, 2006)	1998 (1991, 2005)
Father’s birth year, median (1st and 3rd quartiles)	1964 (1957, 1971)	1965 (1958, 1972)	1967 (1961, 1974)	1966 (1960, 1975)
Mother’s birth year, median (1st and 3rd quartiles)	1966 (1960, 1973)	1968 (1962, 1975)	1970 (1964, 1977)	1970 (1963, 1978)
Paternal immigration status, *N* (%)				
Born in Sweden	2,260,725 (84.1)	172,654 (73.2)	14,608 (76.9)	8,564 (72.7)
Born outside Sweden	428,822 (15.9)	63,091 (26.8)	4,367 (23.0)	3,208 (27.2)
Missing data	348 (0.01)	57 (0.02)	16 (0.1)	5 (0.04)
Maternal immigration status, *N* (%)				
Born in Sweden	2,272,747 (84.5)	183,759 (77.9)	14,665 (77.2)	9,098 (77.3)
Born outside Sweden	416,997 (15.5)	52,038 (22.1)	4,324 (22.8)	2,676 (22.7)
Missing data	151 (0.01)	5 (0.00)	2 (0.01)	3 (0.03)
Highest parental education, *N* (%)				
Primary and lower secondary education	81,914 (3.1)	18,322 (7.8)	1,669 (8.8)	2,138 (18.2)
Upper secondary education	1,083,795 (40.3)	140,211 (59.5)	11,644 (61.3)	7,626 (64.8)
Post-secondary education	1,439,067 (53.5)	75,702 (32.1)	5,507 (29.0)	1,977 (16.8)
Post-graduate education	75,981 (2.8)	1,205 (0.5)	143 (0.8)	11 (0.1)
Missing data	9,138 (0.3)	362 (0.2)	28 (0.2)	25 (0.2)
Any paternal psychiatric disorder, *N* (%)	160,284 (6.0)	64,379 (27.3)	3,098 (16.3)	5,230 (44.4)
Any maternal psychiatric disorder, *N* (%)	233,585 (8.7)	41,780 (17.7)	7,706 (40.6)	5,999 (50.9)
Paternal externalizing disorder, *N* (%)	57,545 (2.1)	42,080 (17.9)	1,717 (9.04)	4,088 (34.7)
Maternal externalizing disorder, *N* (%)	43,636 (1.6)	14,529 (6.2)	3,932 (20.7)	3,952 (33.4)
Paternal suicidal behavior, *N* (%)	43,316 (1.6)	21,294 (9.0)	872 (4.6)	1,897 (16.1)
Maternal suicidal behavior, *N* (%)	59,340 (2.2)	14,068 (6.0)	3,362 (17.7)	2,658 (22.6)
Paternal incarceration, *N* (%)	75,005 (2.8)	100,253 (42.5)	2,209 (11.6)	7,545 (64.1)
Maternal incarceration, *N* (%)	3,993 (0.2)	2,783 (1.2)	1,636 (8.6)	2,389 (20.3)
Father-child coresiding*, *N* (%)				
Father coresiding with the child	2,046,729 (81.9)	116,364 (52.2)	9,504 (52.1)	3,167 (28.0)
Missing data	10,057 (0.4)	788 (0.4)	68 (0.4)	59 (0.5)
Mother–child coresiding*, *N* (%)				
Mother coresiding with the child	2,361,848 (94.5)	205,423 (92.2)	14,812 (81.1)	8,403 (74.4)
Missing data	10,057 (0.4)	788 (0.4)	68 (0.4)	59 (0.5)

*Note:* Information on parental violent convictions, parental incarceration, parental psychiatric disorders, and parental suicidal behavior before the child’s 10th birthday included.

* Indicates whether the child had lived in the same area as his/her parent for at least 8 years of the first 10 years of life. Residential data were obtained from the DeSO (Demographic Statistical Areas) classification, based on which Sweden is divided into 5,984 geographical areas according to county and municipal boundaries, with data updated at the end of each year (Statistics Sweden, 2024). For coresiding information, individuals born in Sweden between 1982 and 2010 (*N* = 2,752,991) were included.

**Table 2.** Cumulative incidence (95% confidence interval) of suicidal behavior by age 30 among offspring with none, one, or two parents convicted of violent offenses

		Offspring of parents without violent convictions (*N* = 2,689,895)	Offspring with one parent convicted of violent offenses (*N* = 254,793)	Offspring with both parents convicted of violent offenses (*N* = 11,777)
		Cumulative incidence, % (95% CI)	Cumulative incidence, % (95% CI)	Cumulative incidence, % (95% CI)
Female offspring				
Any suicidal behavior		4.5 (4.5–4.6)	9.2 (9.0–9.4)	16.6 (15.3–18.0)
Nonfatal self-harm		4.4 (4.4–4.5)	9.1 (8.9–9.3)	16.3 (15.0–17.8)
Suicide		0.1 (0.1–0.1)	0.3 (0.3–0.3)	0.8 (0.5–1.2)
Male offspring				
Any suicidal behavior		4.0 (4.0–4.1)	8.4 (8.2–8.6)	14.3 (13.0–15.7)
Nonfatal self-harm		3.8 (3.8–3.9)	8.0 (7.8–8.2)	13.8 (12.5–15.2)
Suicide		0.3 (0.3–0.3)	0.7 (0.6–0.7)	0.9 (0.6–1.4)

Adjusted population-level associations are shown in Supplementary Table S4. Parental violent convictions were associated with an increased risk for offspring suicidal behavior, with the highest elevated risk seen for those with both parents convicted (HRs 1.96 [95% CI, 1.91–2.02] and 3.37 [95% CI, 3.06–3.71] for male offspring and 1.97 [95% CI, 1.92–2.02] and 3.59 [95% CI, 3.30–3.91] for female offspring with one or two convicted parents, respectively). When separating the outcome of offspring nonfatal self-harm and suicide, similar patterns were observed, but associations appeared somewhat stronger for offspring suicide.

**Figure 1.**
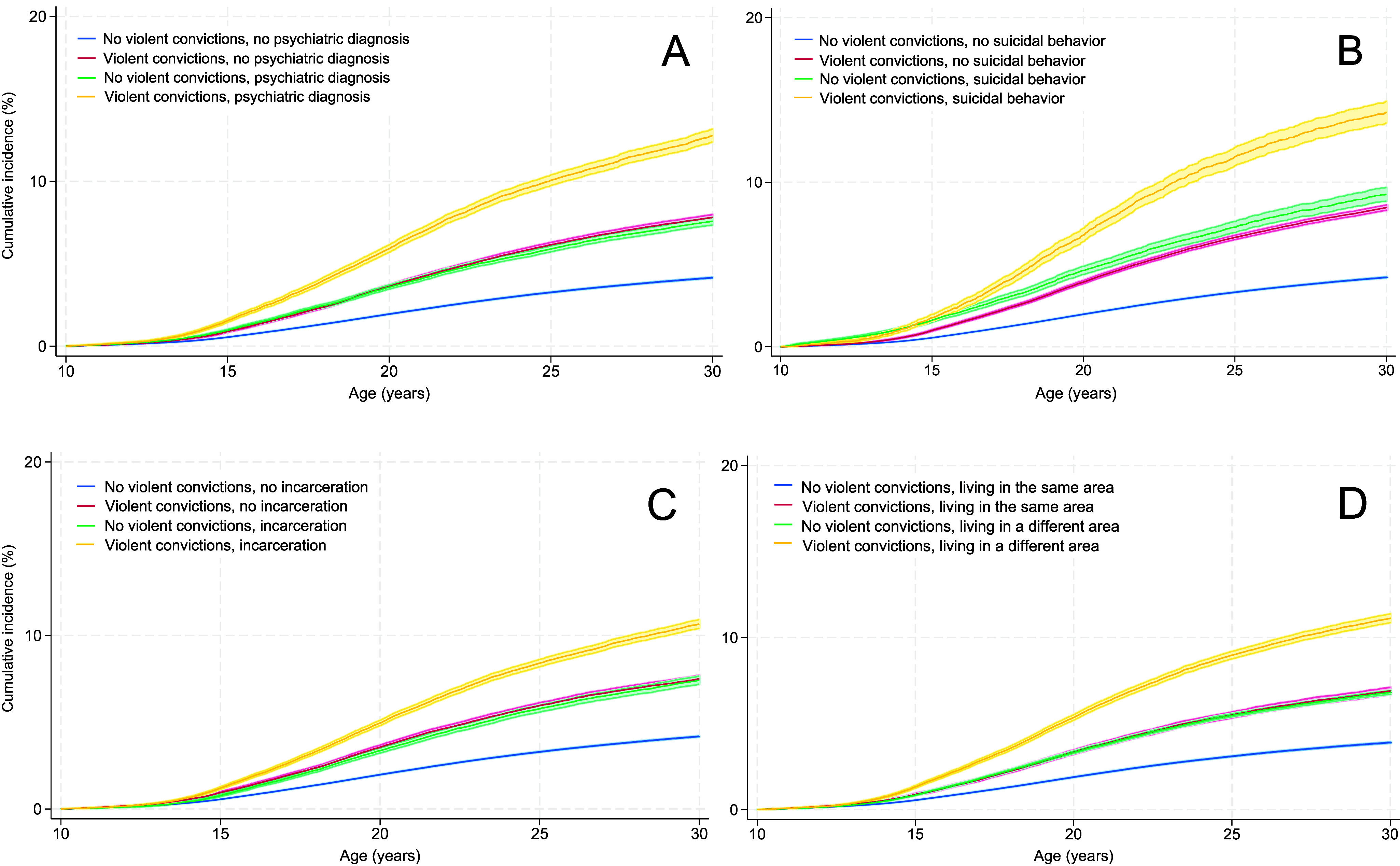
Kaplan–Meier curves for suicidal behavior from age 10 to age 30 among offspring of fathers with/without violent convictions, further stratified by (a) paternal psychiatric disorders, (b) paternal suicidal behavior, (c) paternal incarceration, and (d) the child’s and father’s coresiding.

**Figure 2.**
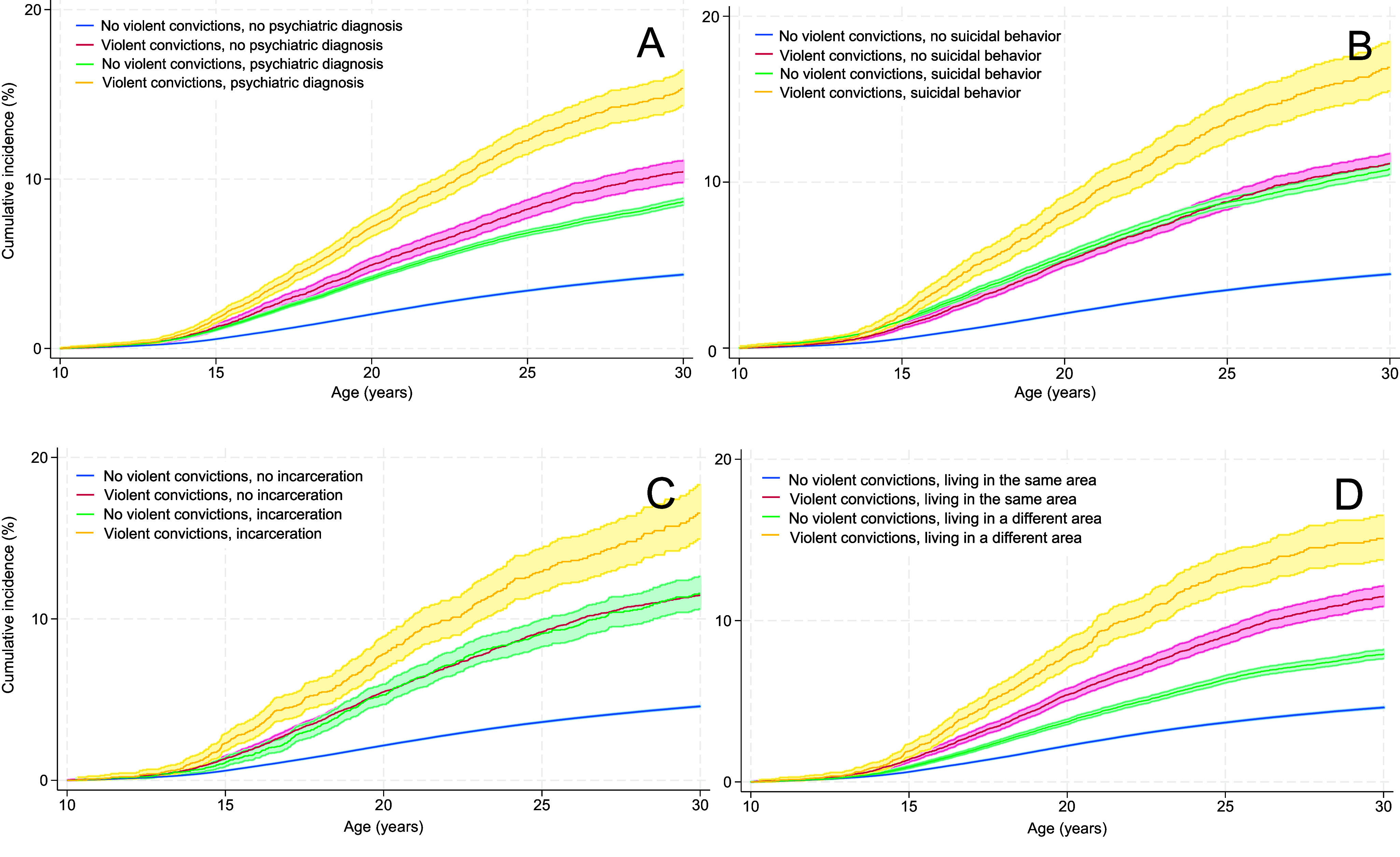
Kaplan–Meier curves for suicidal behavior from age 10 to age 30 among offspring of mothers with/without violent convictions, further stratified by (a) maternal psychiatric disorders, (b) maternal suicidal behavior, (c) maternal incarceration, and (d) the child’s and mother’s coresiding.

We observed a stepwise increase in the absolute risk of suicidal behavior with the number of parental convictions, with the highest risk among offspring of parents with five or more convictions (Supplementary Table S5). Specifically, 13.0% of male and 14.8% of female offspring with a father convicted of five or more violent crimes engaged in suicidal behavior by age 30. The corresponding proportions for offspring of convicted mothers were 19.5% and 15.0%, respectively. As for relative risks, associations between parental violent convictions and offspring suicidal behavior were observed regardless of the number of convictions, but were most pronounced among offspring of parents with five or more convictions (Supplementary Table S6). When analyzing offspring nonfatal self-harm and suicide separately, associations appeared stronger for offspring suicide. Figures 1 and 2 show the cumulative incidence of suicidal behavior from age 10 to age 30 among individuals with and without a convicted father/mother, further stratified by paternal/maternal psychiatric disorders (A), paternal/maternal suicidal behavior (B), paternal/maternal incarceration (C), and the child’s and father’s/mother’s coresiding (D). The exact cumulative incidence rates are given in Supplementary Tables S7 and S8. A similar pattern was found in all stratified models: highest absolute risks were found among offspring whose father/mother had a violent conviction and additionally a psychiatric diagnosis, and the absolute risk was consistently higher in relation to maternal as compared to paternal convictions (12.8% and 15.4%, respectively; *p*-value for the difference in estimates <.001), a healthcare contact of suicidal behavior (14.3% and 16.9%, respectively; *p* = .001), a prison sentence (10.7% and 16.6%, respectively; *p* < .001), or had resided less with the child (11.1% and 15.1%, respectively; *p* < .001), while offspring of parents with only a violent conviction or one of the above-mentioned factors had a more moderately elevated risk. Supplementary Table S9 gives descriptive information on offspring with a father (*n* = 247,579 [8.4%]) or a mother (*n* = 30,768 [1.0%]) convicted of violent offenses.

As for relative risks, parental violent convictions were more strongly associated with suicidal behavior when the father/mother had no history of psychiatric disorders, externalizing disorders, suicidal behavior or incarceration, and when the parent had resided longer with the child as compared to when the parent had psychiatric disorders, suicidal behavior or incarceration, or had a shorter coresidence with the child (Supplementary Table S10). The associations appeared to be generally somewhat stronger for maternal than for paternal violent convictions. Separate analyses of offspring nonfatal self-harm and suicide revealed similar patterns but suggested stronger associations with suicide relative to nonfatal self-harm.

In multivariable models, the independent contributions of individual parental factors to the risk of suicidal behavior were moderate but statistically significant (Supplementary Table S11). No single factor emerged as particularly dominant. The overall picture was broadly similar in analyses conducted separately for offspring nonfatal self-harm and suicide. In analyses examining the accumulation of parental factors, we found that the cumulative incidence of suicidal behavior increased the more parental factors, besides violent convictions, the child was exposed to (Supplementary Figures S2 and S3). The hazard ratios ranged from 1.58 to 4.65 and from 2.13 to 4.20 for offspring of convicted fathers and mothers, respectively, as compared to unexposed offspring (Supplementary Table S12).

The children-of-siblings analyses, presented in Table 3, showed a clear pattern of gradually weaker associations towards models accounting for more genetic influences shared by children of siblings. For example, the minimally adjusted population-level association between paternal convictions and offspring suicidal behavior (HR 2.04 [95% CI, 2.00–2.08]) attenuated in offspring of half-siblings (HR 1.57 [95% CI, 1.47–1.68], p-value for the difference in estimates <.001) and full siblings (HR 1.46 [95% CI, 1.38–1.54], p-value for the difference in estimates <.001) models. In the children of MZ twins model, the estimate was negative but imprecise (HR 0.46 [95% CI, 0.16–1.28], p-value for the difference in estimates .004).

**Table 3.** Associations between paternal/maternal violent convictions and offspring suicidal behavior in the full population and the within-family models with three types of sibling parents with increasing genetic relatedness

	Full population	Within-family models		
		Children of half-siblings	Children of full siblings	Children of MZ twins
	HR (95% CI)	HR (95% CI)	HR (95% CI)	HR (95% CI)
Paternal violent convictions	*N* = 2,956,465	*N* = 531,649	*N* = 1,834,323	*N* = 7,345
Model 1	2.04 (2.00–2.08)	1.57 (1.47–1.68)	1.46 (1.38–1.54)	0.46 (0.16–1.28)
Model 2	1.58 (1.55–1.61)	1.33 (1.24–1.42)	1.29 (1.22–1.37)	0.42 (0.14–1.25)
Maternal violent convictions	*N* = 2,956,465	*N* = 599,088	*N* = 1,877,591	
Model 1	2.50 (2.39–2.62)	1.73 (1.53–1.95)	1.46 (1.30–1.65)	*–*
Model 2	1.54 (1.47–1.62)	1.44 (1.27–1.63)	1.24 (1.10–1.40)	*–*

*Note:* The models for children of half-siblings, full siblings, and MZ twins rule out 25%, 50%, and 100% of the genetic influences shared between the convicted parent and his/her child, respectively. Model 1: adjusted for child’s and parent’s birth years, and parental immigration status. Missing information on parental immigration was treated as a separate category. Model 2: adjusted for child’s and parent’s birth years, parental immigration status, highest parental education, parental psychiatric disorders, and co-parent’s violent criminality. Missing information on parental education was treated as a separate category. *P*-values for the difference between population-level and children of half-siblings estimates <.001 (paternal sibling pairs) and <.001 (maternal sibling pairs), children of half-siblings and children of full-siblings estimates 0.10 (paternal sibling pairs) and 0.06 (maternal sibling pairs), and children of full siblings and children of MZ twins estimates 0.03 (paternal sibling pairs). Models for children of maternal MZ twins are not presented due to highly imprecise estimates. HR = hazard ratio, CI = confidence interval.

As for sensitivity analyses, we found a slight decrease in cumulative incidence rates when events and deaths of undetermined intent were excluded (Supplementary Table S13), while parental violent offending was somewhat more strongly associated with the more narrowly defined concept of suicidal behavior, with some exceptions (Supplementary Table S14). The analysis restricted to offspring of parents with complete information on the exposure yielded estimates similar to those obtained in the original cohort (Supplementary Table S15). Similarly, there was little difference in the estimates from analyses where exposure included all parental violent convictions before the child’s 30th birthday compared to analyses that only included convictions before age 10 (Supplementary Table S16).

## Discussion

This population-based study deepens the current understanding of the risk of suicidal behavior among offspring of parents with violent offending. Using a nationwide cohort of nearly three million individuals, we described the risk of suicidal behavior among offspring with either or both parents convicted of violent crimes, and further promoted the identification of high-risk subgroups by examining the risk in the presence of other parental factors. We also contributed to a better understanding of the nature of the association through a genetically-informed design.

We found a notable incidence of suicidal behavior among offspring of parents with violent offending. Around 14.5% of male and 16.5% of female offspring with both parents convicted of violent offenses engaged in suicidal behavior by age 30. Among offspring with one parent convicted, the corresponding proportions were around 8% and 9%, respectively. In comparison, around 4%–4.5% of children whose parents were not convicted engaged in suicidal behavior by age 30. We also found that the incidence of offspring suicidal behavior increased in relation to the number of parental violent convictions. From a public health perspective, children of parents with violent offending are a group at high risk of suicidal behavior, highlighting the importance of identifying them around the time their parents come into contact with the criminal justice system in order to enable services to identify needs and risks. This could allow for early intervention, which will mitigate longer term risks of suicidal outcomes.

We further examined the absolute risk of suicidal behavior among offspring of parents with criminal convictions in the context of other parental factors indicative of adverse circumstances. Compared to those who were exposed only to violent parental convictions, offspring’s suicidal behavior was even more prevalent when parental violent crime occurred together with parental psychiatric morbidity, externalizing disorders, suicidal behavior, incarceration, or when the convicted parent had resided less with the child. Moreover, the more these factors accumulate in families with parental violent offending, the higher the risk of offspring suicidal behavior. As knowledge about the presence of these factors clearly adds value to the risk assessment of suicidal behavior in the offspring of parents with violent offending, it is important to map the situation of these families in more detail. Such risk stratification could allow for targeting those at greatest risk, while also allowing for more efficient resource allocation. Targeting interventions is likely to require multiagency involvement and collaboration as the identified risks may require addressing from health, social, education, and possibly other agencies.

We further found that the absolute risk of suicidal behavior was systematically higher when offspring were exposed to maternal as compared to paternal violent offending. Maternal criminality may pose a more serious risk to offspring’s well-being, as mothers are often the primary caregivers (Cumming et al., 2023). On the other hand, violent offending was much less common in mothers than in fathers, and in a significant proportion of families where mothers were convicted of violent offenses, fathers also had convictions. Thus, rather than a direct effect of maternal violent offending, families with maternal violent offending may be a highly selected group with particularly poor outcomes for offspring, indicating that children of convicted mothers likely require more detailed assessment, support, and management.

As for relative risks, violent offending of one or both parents was associated with an approximately 2- and 3.5-fold increased risk of suicidal behavior in offspring compared to unexposed children, respectively. Similar population-level associations have been found in previous studies (Björkenstam, Kosidou, & Björkenstam, 2017; O’Hare et al., 2022). Interestingly, parental violent criminality was a weaker predictor of suicidal behavior in offspring whose parent had a record of psychiatric disorder, externalizing disorder, suicidal behavior, or a prison sentence, or when the parent had resided less with the child, as compared to when the parent did not have any of these other factors. This was also evident in multivariable analyses that included parental violent convictions along with the other parental factors, showing statistically significant but moderate independent associations of these factors with offspring suicidal behavior. This is consistent with the interpretation that even though parental violent criminality is associated with an elevated risk of offspring suicidal behavior in all families, its relative importance is lower in families with accumulated adversity compared to those where other adversities are not present to the same extent. In support, a previous study found that the population-level association between maternal incarceration and offspring self-harm weakened when accounting for other adversities, such as maternal mental illness (Cumming et al., 2023). The current findings add to the understanding of the complex interaction of different risk factors, indicating that these factors may not strongly contribute to offspring suicidal behavior independently, but their presence together may point to a highly selected group of families with accumulated genetic and environmental vulnerabilities. In practice, these factors can help to identify children who are particularly prone to suicidal behavior.

When examining offspring nonfatal self-harm and suicide separately, parental violent convictions appeared to be slightly more strongly associated with offspring suicide than with offspring nonfatal self-harm. Parental violent offending may act as an indicator of accumulated difficulties, escalating hardship, genetic factors, and particularly limited coping strategies in such families, which can be reflected in a relatively higher risk for the most serious forms of suicidal behavior. This finding contrasts with the results of an Australian population-based study, which found associations between maternal incarceration and offspring self-harm but not with offspring suicide (Cumming et al., 2023). However, the previous study was likely underpowered to detect the association due to the small number of suicides. While our study indicates a higher relative risk of offspring suicide compared with offspring nonfatal self-harm, few studies in the field have separated out these outcomes that are known to have different risk markers (Favril, Yu, Geddes, & Fazel, 2023), and the topic warrants further investigation.

We further investigated the nature of the intergenerational association and found that the association attenuated as more genetic influences were taken into account, suggesting that genetic predispositions play a role in the link. This is supported by previous studies finding violence and self-harm to be associated at the individual level (O’Donnell et al., 2015), with genetic predisposition to traits associated with aggression and impulsivity being one possible mechanism driving the link (Mann et al., 2009). Unmeasured factors unique to each nuclear family may also play a role, but we were unable to estimate their contribution as the study design is only able to account for factors that are constant within the extended family clusters (D’Onofrio, Lahey, Turkheimer, & Lichtenstein, 2013). However, our results do not exclude the possibility of a causal link, albeit weaker than the population-level associations suggest. Future research with stronger quasi-experimental designs is needed to fully address the relative contributions of unmeasured confounds and the potential impact of parental violent crime.

The results should be interpreted in light of several limitations. First, as we had access only to outcomes in specialized care, our findings relate to the more severe forms of self-harm. This is considered a limitation given that a proportion of self-harm-related events end up in hospital care and in registers (Hawton, Saunders, & O’Connor, 2012; Sahlin et al., 2017). It is probable that we have captured different levels of suicidal behavior depending on the age of offspring, as, for example, less severe self-harm is more pronounced among adolescents than in adults (Lee et al., 2019; Parellada et al., 2008). Second, we had no data on parental violence outside the official registers, leaving less severe violent acts untested. Third, we had no information on the child’s contact with child protection services and decisions on out-of-home care, which are fairly common among children of parents with criminal offending and have also been associated with an increased risk of self-harm (Cumming et al., 2023). We also could not distinguish whether parental violence was domestic in nature, being thus unable to assess the role of child maltreatment in this context. Finally, we assume that the findings are primarily generalizable to countries with similar social structures and legal, social, and health care systems to Sweden.

In conclusion, our findings identify offspring of parents with violent convictions as a high-risk population for suicidal behavior, who could benefit from early identification and multiagency coordination, particularly between social and health services. The prevalence of suicidal behavior is particularly high among those whose mother or both parents have been convicted, and when other family adversity is present, highlighting the need to carefully monitor these offspring. The intergenerational association of these risks is partly explained by genetic factors shared by parents and their offspring, which may help in designing and targeting best practices to support these children.

## Supporting information

10.1017/S0033291726103717.sm001 Järvinen et al. supplementary material Järvinen et al. supplementary material

## Data Availability

Due to the nature of the data, they cannot be made publicly available. Researchers interested in accessing the data can apply for permission to access the register data.

## Supplementary material

The supplementary material for this article can be found at http://doi.org/10.1017/S0033291726103717.
