# Supplementary material for: Parental violent offending and offspring suicidal behavior: a nationwide register-based study
Source: Psychol Med. 2026 Apr 1;56:e87. doi: 10.1017/S0033291726103717 (PMC13079218; doi:10.1017/S0033291726103717)
Supplement: Järvinen et al. supplementary material [file S0033291726103717sup001.docx]

**Supplementary Material for Järvinen A, Fazel S, Kuja-Halkola R, Brikell I, Chang Z, D’Onofrio B M, Larsson H, Lichtenstein P, Latvala A.: Parental violent offending and offspring suicidal behavior: A nationwide register-based study**

**Table of Contents**

[**Appendix S1.** Supplementary Methods 1](#_Toc214009678)

[**Figure S1.** Family pedigrees for children of MZ twins, children of full siblings/DZ twins, and children of half-siblings. 2](#_Toc214009679)

[**Table S1.** List of registers used in the study. 3](#_Toc214009680)

[**Table S2.** Convictions for violent and sexual offenses. 3](#_Toc214009681)

[**Table S3.** ICD-8/9 and ICD-10 codes for variables used in the study. 4](#_Toc214009682)

[**Table S4.** Results from Cox regression analyses for the population-level associations between parental violent convictions and offspring suicidal behavior. 5](#_Toc214009683)

[**Table S5.** Cumulative incidence of suicidal behavior by age 30 among offspring with a father/mother convicted of violent offenses, with the exposure stratified by the number of convictions. 6](#_Toc214009684)

[**Table S6.** Results from Cox regression analysis for the population-level associations between parental violent convictions and offspring suicidal behavior, with the exposure stratified by the number of convictions. 7](#_Toc214009685)

[**Table S7.** Cumulative incidence of suicidal behavior by age 30 among offspring with/without father convicted of violent offenses, further stratified by information on other parental factors. 8](#_Toc214009686)

[**Table S8.** Cumulative incidence of suicidal behavior by age 30 among offspring with/without mother convicted of violent offenses, further stratified by information on other parental factors. 9](#_Toc214009687)

[**Table S9.** Characteristics of offspring born between 1977 and 2010 with fathers and mothers with and without violent convictions. 10](#_Toc214009688)

[**Table** **S10.** Associations between parental violent convictions and offspring suicidal behavior, stratified by information on other parental factors. 12](#_Toc214009689)

[**Table S11.** Multivariable Cox regression models for the associations of parental violent offending and other parental factors with offspring suicidal behavior. 13](#_Toc214009690)

[**Figures S2-S3.** Kaplan-Meier curves estimating the cumulative incidence of suicidal behavior for offspring unexposed to paternal/maternal violent convictions and other parental factors. 14](#_Toc214009691)

[**Table S12.** Results from Cox regression analyses for the association between paternal/maternal violent convictions and other adversities and offspring suicidal behavior, with exposure stratified by the number of parental factors to which the child had been exposed. 15](#_Toc214009692)

[**Table S13.** Cumulative incidence of suicidal behavior (with events and deaths of undetermined intent excluded) by age 30 among offspring with none, one, or two parents convicted. 15](#_Toc214009693)

[**Table S14.** Results from Cox regression analyses for the population-level associations between parental violent convictions and offspring suicidal behavior, with events and deaths of undetermined intent excluded. 16](#_Toc214009694)

[**Table S15.** Results from Cox regression analyses for the population-level associations between parental violent convictions and offspring suicidal behavior, with the sample restricted to children whose parents were born from 1958 onwards. 16](#_Toc214009695)

[**Table S16.** Results from Cox regression analyses for the population-level associations between parental violent convictions and offspring suicidal behavior, with exposure including all parental violent convictions before the child's 30th birthday. 16](#_Toc214009696)

[**References** 17](#_Toc214009697)

# **Appendix S1.** **Supplementary Methods**

Study population

Information on twin parents and their zygosity was obtained from the Swedish Twin Registry (Zagai et al., 2019). Within-family analyses included 531,649 and 599,088 observations from paternal/maternal half-siblings, 1,834,323 and 1,877,591 observations from paternal/maternal full siblings or DZ twins, and 7,345 and 9,178 observations from paternal/maternal MZ twins, respectively. The same individual could appear both in a family identified through the father and in a family identified through the mother. Further, in the models for children of half-siblings, same individuals could appear more than once in each model if they belonged to more than one extended family cluster identified through half-sibling parents.

Exposure

In main analyses, we defined parental violent offending as a three-level variable with mutually exclusive categories indicating criminal convictions for violent (including sexual) offenses of none, one, or two parents before the child's 10^th^ birthday, including convictions also before the child’s birth. We further created a variable indicating the number of parental violent convictions during the first 10 years of the child’s life, including four mutually exclusive categories (0, 1, 2–4, 5 or more convictions). For stratified analyses and within-family analyses, we defined the exposure as a dichotomous variable denoting whether the parent had been convicted of a violent crime at any time before the child’s 10^th^ birthday, separately for fathers and mothers.

Information about convictions was obtained from the National Crime Register, which contains data on all criminal convictions and sanctions imposed by district courts on individuals aged 15 (age of criminal responsibility in Sweden) and above since 1973 (Brottsförebyggande rådet, 2024). We used the date of conviction to determine the child’s age at the time of exposure, as there was incomplete information on the timing of offenses. Details of the convictions are given in Table S2.

Statistical analysis

As for within-family analyses, the models for clusters of children of half-siblings, full siblings/DZ twins, and MZ twins were run separately for children identified through the father and through the mother, thus excluding children of opposite-sex sibling pairs from the analyses.

The models compare the risk of suicidal behavior between cousins who are discordant for exposure to parental violent convictions and rule out all genetic and environmental factors that are constant within the clusters. As for genetic factors, the models for children of half-siblings, full siblings/DZ twins, and MZ twins rule out 25%, 50%, and 100% of the genetic influences shared between the convicted parent and his/her child, respectively (Figure S1). For example, in the case of children of full siblings/DZ twins, the unexposed child and his/her convicted uncle/aunt share approximately 25% of their genes, which is 50% of the genetic similarity between the convicted parent and his/her child.


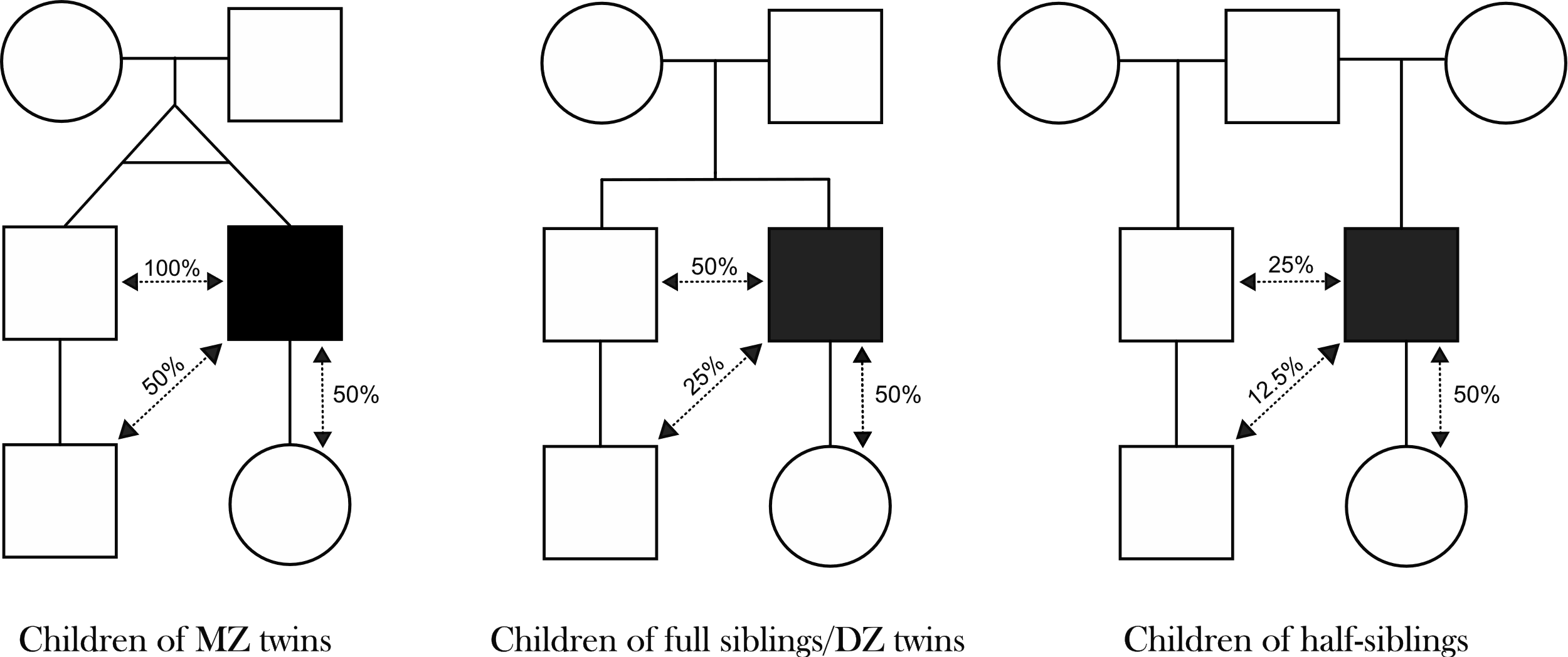


# **Figure S1.** Family pedigrees for children of MZ twins, children of full siblings/DZ twins, and children of half-siblings, including information on the proportion of shared genes. The black square represents parental violent offending. Children identified through the father presented here.

| **Table S1.** List of registers used in the study. | | | | |
| --- | --- | --- | --- | --- |
| **Register** |  | **Information used in the study** |  | **Temporal coverage of the register** |
| The Total Population Register (Statistics Sweden) |  | Basic person data (date of birth, birth country, sex) |  | 1932– |
| The Multi-Generation Register (Statistics Sweden) |  | Biological parents and grandparents |  | 1932– |
| The National Census (Statistics Sweden) |  | Highest education |  | 1970–1990 |
| The Longitudinal Integration Database for Health Insurance and Labor Market Studies (Statistics Sweden) |  | Highest education |  | 1990– |
| The Migration Register (Statistics Sweden) |  | Immigration and emigration |  | 1968– |
| The Swedish Twin Registry |  | Twin parents and their zygosity |  | 1958– |
| The National Crime Register (Swedish National Council for Crime Prevention) |  | Criminal convictions and sanctions imposed by district courts |  | 1973– |
| The National Patient Register (National Board of Health and Welfare) |  | Diagnostic information according to the International Classification of Diseases |  | Inpatient and outpatient diagnoses in specialized healthcare since 1973 and 2001, respectively |
| The Cause of Death Register (National Board of Health and Welfare) |  | Death causes |  | 1952– |
| Demographic Statistical Areas (Statistics Sweden) |  | Demographical data |  | 1982– |

| **Table S2.** Convictions for violent and sexual offenses. | | |
| --- | --- | --- |
| **Offense type** | **Convictions** |  |
| Violent crime | Murder, manslaughter, assault, kidnapping, illegal restraint, illegal coercion or threats, robbery, threats or violence against an officer, arson, gross violation of a person’s integrity, harassment | |
| Sexual crime | Rape, sexual coercion, child molestation, sexual intercourse with a child, child pornography offenses, pimping, sexual harassment | |

| **Table S3.** ICD-8/9 (based on Swedish translations) and ICD-10 codes for variables used in the study. For suicidal behavior, information on nonfatal events was retrieved from the National Patient Register (NPR), and mortality data from the Cause of Death Register. | | |
| --- | --- | --- |
| **Variable** | **ICD-8/9** | **ICD-10** |
| **Suicidal behavior** | |  |
| Any suicidal behavior |  |  |
| Nonfatal self-harm (nonfatal intentional self-harm, events  of undetermined intent) (NPR) | E950–950, E980–989 | X60–84, Y10–34, Y87.2, Y87.0 |
| Suicide (suicide, death of undetermined intent) (Cause of  Death Register) | E950–950, E980–989 | X60–84, Y10–34, Y87.2, Y87.0 |
| Suicidal behavior with events of undetermined intent excluded (sensitivity analysis) |  |  |
| Nonfatal intentional self-harm (NPR) | E950–959 | X60–84, Y87.0 |
| Suicide (Cause of Death Register) | E950–959 | X60–84, Y87.0 |
| **Psychiatric disorders** |  |  |
| Any psychiatric disorder (all mental and behavioral disorders) | 290–319 | F00–99 |
| Organic, including symptomatic, mental disorders | 290, 293, 294, 310 | F00–09 |
| Mental and behavioral disorders due to psychoactive  substance use | 291, 292, 303–305 | F10–19 |
| Schizophrenia, schizotypal and delusional disorders | 295, 297, 298.2–298.9 | F20–29 |
| Mood (affective) disorders | 296, 298.0, 300, 311 | F30–39 |
| Neurotic, stress-related and somatoform disorders | 300, 308, 309 | F40–48 |
| Behavioral syndromes associated with physiological  disturbances and physical factors | 316 | F50–59 |
| Disorders of adult personality and behavior | 301 | F60–69 |
| Mental retardation | 317–319 | F70–79 |
| Disorders of psychological development | 299, 315 | F80–89 |
| Behavioral and emotional disorders with onset usually  occurring in childhood and adolescence | 312–314 | F90–98 |
| Unspecified mental disorder | 298.9, 300.9 | F99 |
| **Externalizing disorders** |  |  |
| ADHD | 314 | F90 |
| Conduct disorder | 312 | F91 |
| Antisocial personality disorder | 301.7 | F60.2 |
| Substance use disorders | 291, 292, 303–305 | F10–19 |

| **Table S4.** Results from Cox regression analyses for the population-level associations (N=2,956,465) between parental violent convictions and offspring suicidal behavior. | | | | |
| --- | --- | --- | --- | --- |
|  |  | **Male offspring** |  | **Female offspring** |
|  |  | HR (95% CI) |  | HR (95% CI) |
| **Any suicidal behavior** | |  |  |  |
| One parent with violent convictions | | 1.96 (1.91–2.02) |  | 1.97 (1.92–2.02) |
| Two parents with violent convictions | | 3.37 (3.06–3.71) |  | 3.59 (3.30–3.91) |
| **Nonfatal self-harm** | |  |  |  |
| One parent with violent convictions | | 1.96 (1.91–2.02) |  | 1.97 (1.92–2.02) |
| Two parents with violent convictions | | 3.41 (3.09–3.76) |  | 3.58 (3.28–3.90) |
| **Suicide** | |  |  |  |
| One parent with violent convictions | | 2.22 (2.00–2.48) |  | 2.23 (1.88–2.63) |
| Two parents with violent convictions | | 3.27 (2.19–4.90) |  | 6.28 (4.02–9.81) |
| Adjusted for child's and parent's birth years, and parental immigration status. Missing information on parental immigration was treated as a separate category. HR=hazard ratio, CI=confidence interval | | | | |

| **Table S5.** Cumulative incidence (95% confidence interval) of suicidal behavior by age 30 among offspring with a father/mother convicted of violent offenses, with the exposure further stratified by the number of convictions. | | | | | | | | | | | | | |
| --- | --- | --- | --- | --- | --- | --- | --- | --- | --- | --- | --- | --- | --- |
|  |  | | **Any suicidal behavior** | | |  | **Nonfatal self-harm** | | |  | **Suicide** | | |
|  |  | **Male offspring** | |  | **Female offspring** |  | **Male offspring** |  | **Female offspring** |  | **Male offspring** |  | **Female offspring** |
|  |  | Cumulative incidence (95% CI) | |  | Cumulative incidence (95% CI) |  | Cumulative incidence (95% CI) |  | Cumulative incidence (95% CI) |  | Cumulative incidence (95% CI) |  | Cumulative incidence (95% CI) |
| **Paternal violent convictions** | | | |  |  |  |  |  |  |  |  |  |  |
| No convictions | | | 4.2 (4.2–4.2) |  | 4.7 (4.7–4.8) |  | 4.0 (3.9–4.0) |  | 4.6 (4.6–4.7) |  | 0.3 (0.3–0.3) |  | 0.1 (0.1–0.1) |
| One conviction | | | 8.8 (8.4–9.3) |  | 9.8 (9.3–10.3) |  | 8.4 (7.9–8.8) |  | 9.7 (9.3–10.2) |  | 0.7 (0.5–0.8) |  | 0.2 (0.2–0.3) |
| 2–4 convictions | | | 10.4 (9.8–11.1) |  | 11.4 (10.8–12.0) |  | 9.9 (9.4–10.5) |  | 11.2 (10.6–11.9) |  | 0.8 (0.6–1.0) |  | 0.5 (0.3–0.6) |
| 5 convictions or more | | | 13.0 (11.9–14.1) |  | 14.8 (13.8–16.0) |  | 12.4 (11.4–13.5) |  | 14.7 (13.6–15.8) |  | 1.1 (0.8–1.5) |  | 0.5 (0.3–0.9) |
| **Maternal violent convictions** | | | | | |  |  |  |  |  |  |  |  |
| No convictions | | | 4.4 (4.3–4.4) |  | 4.9 (4.9–5.0) |  | 4.2 (4.1–4.2) |  | 4.8 (4.8–4.9) |  | 0.3 (0.3–0.3) |  | 0.1 (0.1–0.1) |
| One conviction | | | 11.4 (10.1–12.8) |  | 13.4 (12.0–14.8) |  | 11.0 (9.8–12.4) |  | 13.1 (11.8–14.5) |  | 0.9 (0.6–1.5) |  | 0.9 (0.6–1.5) |
| 2–4 convictions | | | 14.0 (11.7–16.7) |  | 14.1 (11.9–16.6) |  | 13.3 (11.0–15.9) |  | 13.8 (11.7–16.4) |  | 1.2 (0.6–2.4) |  | 0.7 (0.3–1.6) |
| 5 convictions or more | | | 19.5 (12.3–30.1) |  | 15.0 (10.1–22.1) |  | 18.3 (11.2–29.0) |  | 15.0 (10.1–22.1) |  | 1.5 (0.4–5.7) |  | 0.9 (0.1–5.9) |
| Sample sizes for each stratum: offspring of fathers without convictions (N = 1,466,739 males, 1,386,932 females), with one (N = 27,750 males, 25,970 females), 2–4 (N = 18,332 males, 17,462 females), or 5 or more convictions (N = 6,931 males, 6,349 females); offspring of mothers without convictions (N = 1,512,824 males, 1,430,379 females), with one (N = 4,858 males, 4,408 females), 2–4 (N = 1,788 males, 1,649 females), or 5 or more convictions (N = 282 males, 277 females) | | | | | | | | | | | | | |

| **Table S6.** Results from Cox regression analysis for the population-level associations between parental violent convictions and offspring suicidal behavior, with the exposure stratified by the number of convictions that occurred during the child's first 10 years of life. Reference category: offspring of parents without violent convictions. | | | | | | | | | | | | |
| --- | --- | --- | --- | --- | --- | --- | --- | --- | --- | --- | --- | --- |
|  |  | **Any suicidal behavior** | | |  | **Nonfatal self-harm** | | |  | **Suicide** | | |
|  |  | **Male offspring** |  | **Female offspring** |  | **Male offspring** |  | **Female offspring** |  | **Male offspring** |  | **Female offspring** |
|  |  | HR (95% CI) |  | HR (95% CI) |  | HR (95% CI) |  | HR (95% CI) |  | HR (95% CI) |  | HR (95% CI) |
| **Paternal violent convictions** | |  |  |  |  |  |  |  |  |  |  |  |
| One conviction | | 2.04 (1.93–2.15) |  | 2.04 (1.94–2.14) |  | 2.03 (1.93–2.15) |  | 2.04 (1.94–2.15) |  | 2.12 (1.72–2.61) |  | 1.91 (1.36–2.68) |
| 2–4 convictions | | 2.40 (2.26–2.55) |  | 2.38 (2.25–2.51) |  | 2.40 (2.25–2.55) |  | 2.37 (2.24–2.51) |  | 2.48 (1.96–3.14) |  | 3.59 (2.64–4.90) |
| 5 convictions or more | | 2.95 (2.71–3.22) |  | 3.13 (2.89–3.39) |  | 2.95 (2.70–3.22) |  | 3.14 (2.90–3.40) |  | 3.62 (2.66–4.94) |  | 3.86 (2.41–6.17) |
| **Maternal violent convictions** | | |  |  |  |  |  |  |  |  |  |  |
| One conviction | | 2.38 (2.12–2.68) |  | 2.55 (2.30–2.84) |  | 2.41 (2.14–2.71) |  | 2.54 (2.28–2.83) |  | 2.58 (1.64–4.05) |  | 5.50 (3.40–8.91) |
| 2–4 convictions | | 3.09 (2.59–3.69) |  | 2.86 (2.41–3.39) |  | 3.06 (2.55–3.67) |  | 2.85 (2.40–3.38) |  | 3.74 (1.94–7.20) |  | 4.96 (2.06–11.95) |
| 5 convictions or more | | 3.15 (1.99–5.01) |  | 3.23 (2.14–4.86) |  | 2.92 (1.79–4.76) |  | 3.61 (2.17–4.91) |  | 6.06 (1.51–24.24) |  | 5.99 (0.84–42.58) |
| Adjusted for child's and parent's birth years, and parental immigration status. Missing information on parental immigration was treated as a separate category.  Sample sizes for each stratum: offspring of fathers without convictions (N = 1,466,739 males, 1,386,932 females), with one (N = 27,750 males, 25,970 females), 2–4 (N = 18,332 males, 17,462 females), or 5 or more convictions (N = 6,931 males, 6,349 females); offspring of mothers without convictions (N = 1,512,824 males, 1,430,379 females), with one (N = 4,858 males, 4,408 females), 2–4 (N = 1,788 males, 1,649 females), or 5 or more convictions (N = 282 males, 277 females) HR=hazard ratio, CI=confidence interval | | | | | | | | | | | | |

| **Table S7.** Cumulative incidence (95% confidence interval) of suicidal behavior by age 30 among offspring with/without father convicted of violent offenses, further stratified by information on other parental factors. | | | | | | | | | | | |
| --- | --- | --- | --- | --- | --- | --- | --- | --- | --- | --- | --- |
|  | **Any suicidal behavior** | | |  | **Nonfatal self-harm** | | |  | **Suicide** | | |
|  | **Offspring with father not convicted of violent offenses** |  | **Offspring with father convicted of violent offenses** |  | **Offspring with father not convicted of violent offenses** |  | **Offspring with father convicted of violent offenses** |  | **Offspring with father not convicted of violent offenses** |  | **Offspring with father convicted of violent offenses** |
|  | Cumulative incidence,  % (95% CI) |  | Cumulative incidence,  % (95% CI) |  | Cumulative incidence,  % (95% CI) |  | Cumulative incidence,  % (95% CI) |  | Cumulative incidence,  % (95% CI) |  | Cumulative incidence,  % (95% CI) |
| **Paternal psychiatric morbidity** |  |  |  |  |  |  |  |  |  |  |  |
| No psychiatric diagnosis | 4.2 (4.1–4.2) |  | 7.8 (7.6–8.0) |  | 4.0 (4.0–4.1) |  | 7.6 (7.4–7.8) |  | 0.2 (0.2–0.2) |  | 0.4 (0.4–0.5) |
| Psychiatric diagnosis | 7.6 (7.4–7.8) |  | 12.8 (12.4–13.2) |  | 7.3 (7.1–7.5) |  | 12.4 (12.0–12.8) |  | 0.5 (0.4–0.6) |  | 0.7 (0.6–0.9) |
| **Paternal externalizing disorder** |  |  |  |  |  |  |  |  |  |  |  |
| No externalizing disorder | 4.2 (4.2–4.3) |  | 8.0 (7.9–8.2) |  | 4.1 (4.1–4.1) |  | 7.8 (7.6–7.9) |  | 0.2 (0.2–0.2) |  | 0.4 (0.4–0.5) |
| Externalizing disorder | 8.9 (8.6–9.3) |  | 13.9 (13.4–14.4) |  | 8.6 (8.2–9.0) |  | 13.5 (13.0–14.0) |  | 0.6 (0.5–0.8) |  | 0.8 (0.7–0.9) |
| **Paternal suicidal behavior** |  |  |  |  |  |  |  |  |  |  |  |
| No suicidal behavior | 4.2 (4.2–4.3) |  | 8.5 (8.3–8.6) |  | 4.1 (4.1–4.1) |  | 8.2 (8.1–8.4) |  | 0.2 (0.2–0.2) |  | 0.4 (0.4–0.5) |
| Suicidal behavior | 9.3 (8.9–9.7) |  | 14.3 (13.6–14.9) |  | 8.9 (8.5–9.3) |  | 13.7 (13.1–14.4) |  | 0.6 (0.5–0.7) |  | 0.9 (0.7–1.1) |
| **Paternal incarceration** |  |  |  |  |  |  |  |  |  |  |  |
| No incarceration | 4.2 (4.2–4.2) |  | 7.5 (7.3–7.7) |  | 4.1 (4.0–4.1) |  | 7.3 (7.1–7.5) |  | 0.2 (0.2–0.2) |  | 0.4 (0.3–0.4) |
| Incarceration | 7.4 (7.2–7.7) |  | 10.7 (10.4–10.9) |  | 7.2 (6.9–7.4) |  | 10.4 (10.1–10.6) |  | 0.4 (0.4–0.5) |  | 0.6 (0.5–0.7) |
| **Child–parent coresiding** |  |  |  |  |  |  |  |  |  |  |  |
| Living in the same area | 3.9 (3.9–3.9) |  | 6.9 (6.7–7.1) |  | 3.8 (3.7–3.8) |  | 6.7 (6.5–6.9) |  | 0.2 (0.2–0.2) |  | 0.4 (0.3–0.4) |
| Living in a different area | 6.8 (6.7–6.9) |  | 11.1 (10.9–11.4) |  | 6.6 (6.5–6.7) |  | 10.8 (10.6–11.1) |  | 0.4 (0.3–0.4) |  | 0.6 (0.6–0.7) |
| Sample sizes for each stratum: Offspring of fathers **without** violent convictions: paternal psychiatric disorder (N = 163,382), no paternal psychiatric disorder (N = 2,545,504); paternal externalizing disorder (N = 59,262), no paternal externalizing disorder (N = 2,649,624); paternal suicidal behavior (N = 44,188), no paternal suicidal behavior (N = 2,664,698); paternal incarceration (N = 77,214), no paternal incarceration (N = 2,631,672); living in the same area (N = 2,067,205), living in a different area (N = 452,495). Offspring of fathers **with** violent convictions: paternal psychiatric disorder (N = 69,609), no paternal psychiatric disorder (N = 177,970); paternal externalizing disorder (N = 46,168), no paternal externalizing disorder (N = 201,411); paternal suicidal behavior (N = 23,191), no paternal suicidal behavior (N = 224,388); paternal incarceration (N = 107,798), no paternal incarceration (N = 139,781); living in the same area (N = 119,531), living in a different area (N = 133,760). | | | | | | | | | | | |

| **Table S8.** Cumulative incidence (95% confidence interval) of suicidal behavior by age 30 among offspring with/without mother convicted of violent offenses, further stratified by information on other parental factors. | | | | | | | | | | | |  |  |  |
| --- | --- | --- | --- | --- | --- | --- | --- | --- | --- | --- | --- | --- | --- | --- |
|  | **Any suicidal behavior** | | |  | **Nonfatal self-harm** | | |  | **Suicide** | | |  |  |  |
|  | **Offspring with mother not convicted of violent offenses** |  | **Offspring with mother convicted of violent offenses** |  | **Offspring with mother not convicted of violent offenses** |  | **Offspring with mother convicted of violent offenses** |  | **Offspring with mother not convicted of violent offenses** |  | **Offspring with mother convicted of violent offenses** |  |  |  |
|  | Cumulative incidence,  % (95% CI) |  | Cumulative incidence,  % (95% CI) |  | Cumulative incidence,  % (95% CI) |  | Cumulative incidence,  % (95% CI) |  | Cumulative incidence,  % (95% CI) |  | Cumulative incidence,  % (95% CI) |  |  |  |
| **Maternal psychiatric morbidity** | | | |  |  |  |  |  |  |  |  |  |  |  |
| No psychiatric diagnosis | 4.4 (4.3–4.4) |  | 10.4 (9.8–11.1) |  | 4.2 (4.2–4.3) |  | 10.1 (9.5–10.7) |  | 0.2 (0.2–0.2) |  | 0.8 (0.6–1.0) |  |  |  |
| Psychiatric diagnosis | 8.7 (8.5–8.9) |  | 15.4 (14.3–16.4) |  | 8.4 (8.2–8.6) |  | 15.0 (14.0–16.0) |  | 0.5 (0.5–0.6) |  | 1.0 (0.7–1.3) |  |  |  |
| **Maternal externalizing disorder** | | | |  |  |  |  |  |  |  |  |  |  |  |
| No externalizing disorder | 4.5 (4.5–4.5) |  | 10.8 (10.2–11.4) |  | 4.4 (4.3–4.4) |  | 10.5 (9.9–11.1) |  | 0.2 (0.2–0.2) |  | 0.7 (0.6–0.9) |  |  |  |
| Externalizing disorder | 12.5 (12.0–13.0) |  | 17.4 (16.1–18.9) |  | 12.1 (11.6–12.6) |  | 16.9 (15.6–18.4) |  | 0.8 (0.6–0.9) |  | 1.3 (0.9–1.9) |  |  |  |
| **Maternal suicidal behavior** |  |  |  |  |  |  |  |  |  |  |  |  |  |  |
| No suicidal behavior | 4.5 (4.4–4.5) |  | 11.1 (10.6–11.7) |  | 4.3 (4.3–4.4) |  | 10.8 (10.2–11.4) |  | 0.2 (0.2–0.2) |  | 0.8 (0.6–1.0) |  |  |  |
| Suicidal behavior | 10.8 (10.5–11.1) |  | 16.9 (15.5–18.5) |  | 10.4 (10.1–10.7) |  | 16.4 (15.0–17.9) |  | 0.7 (0.6–0.8) |  | 1.1 (0.7–1.6) |  |  |  |
| **Maternal incarceration** |  |  |  |  |  |  |  |  |  |  |  |  |  |  |
| No incarceration | 4.6 (4.6–4.6) |  | 11.5 (10.9–12.1) |  | 4.4 (4.4–4.5) |  | 11.1 (10.6–11.7) |  | 0.2 (0.2–0.2) |  | 0.8 (0.7–1.0) |  |  |  |
| Incarceration | 11.6 (10.6–12.6) |  | 16.6 (15.0–18.3) |  | 11.3 (10.4–12.3) |  | 16.0 (14.5–17.8) |  | 0.6 (0.4–1.0) |  | 1.0 (0.6–1.6) |  |  |  |
| **Child–parent coresiding** |  |  |  |  |  |  |  |  |  |  |  |  |  |  |
| Living in the same area | 4.6 (4.6–4.7) |  | 11.5 (10.9–12.2) |  | 4.5 (4.4–4.5) |  | 11.2 (10.6–11.8) |  | 0.2 (0.2–0.2) |  | 0.8 (0.6–1.1) |  |  |  |
| Living in a different area | 7.9 (7.7–8.2) |  | 15.1 (13.8–16.5) |  | 7.7 (7.4–7.9) |  | 14.6 (13.3–16.0) |  | 0.4 (0.4–0.5) |  | 1.2 (0.8–1.8) |  |  |  |
| Sample sizes for each stratum: Offspring of mothers **without** violent convictions: maternal psychiatric disorder (N = 275,365), no maternal psychiatric disorder (N = 2,650,332); maternal externalizing disorder (N = 58,165), no maternal externalizing disorder (N = 2,867,532); maternal suicidal behavior (N = 73,418), no maternal suicidal behavior (N = 2,852,279); maternal incarceration (N = 6,776), no maternal incarceration (N = 2,918,921); living in the same area (N = 2,578,243), living in a different area (N = 145,318). Offspring of mothers **with** violent convictions: maternal psychiatric disorder (N = 13,705), no maternal psychiatric disorder (N = 17,063); maternal externalizing disorder (N = 7,884), no maternal externalizing disorder (N = 22,884); maternal suicidal behavior (N = 6,020), no maternal suicidal behavior (N = 24,748); maternal incarceration (N = 4,025), no maternal incarceration (N = 26,743); living in the same area (N = 23,215), living in a different area (N = 6,215). | | | | | | | | | | | |  |  |  |

| **Table S9.** Characteristics of offspring born between 1977 and 2010 (N=2,956,465) with fathers and mothers with and without violent convictions before the child’s 10th birthday. | | | | | |
| --- | --- | --- | --- | --- | --- |
|  | **Offspring with paternal violent convictions**  (N = 247,579) | **Offspring without paternal violent convictions** (N = 2,708,886) |  | **Offspring with maternal violent convictions** (N = 30,768) | **Offspring without maternal violent convictions**  (N = 2,925,697) |
| **Sex, N (%)** |  |  |  |  |  |
| Men | 127,457 (51.5) | 1,392,295 (51.4) |  | 15,875 (51.6) | 1,503,877 (51.4) |
| Women | 120,122 (48.5) | 1,316,591 (48.6) |  | 14,893 (48.4) | 1,421,820 (48.6) |
| **Birth year, median (1st and 3rd quartiles)** | 1996 (1989, 2004) | 1995 (1989, 2004) |  | 1999 (1991, 2005) | 1995 (1989, 2004) |
| **Father’s birth year, median (1st and 3rd quartiles)** | 1965 (1959, 1972) | 1964 (1957, 1971) |  | 1967 (1960, 1975) | 1964 (1957, 1971) |
| **Mother’s birth year, median (1st and 3rd quartiles)** | 1968 (1962, 1975) | 1966 (1960, 1973) |  | 1970 (1963, 1977) | 1966 (1960, 1973) |
| **Number of parental violent convictions during the child’s first 10 years of life** |  |  |  |  |  |
| No convictions | 144,785 (58.5) | – |  | 17,506 (56.9) | – |
| One conviction | 53,720 (21.7) | – |  | 9,266 (30.1) | – |
| 2–4 convictions | 35,794 (14.5) | – |  | 3,437 (11.2) | – |
| 5 convictions or more | 13,280 (5.4) | – |  | 559 (1.8) | – |
| **Co-parent’s violent convictions, N (%)** | 11,777 (4.8) | 18,991 (0.7) |  | 11,777 (38.3) | 235,802 (8.1) |
| **Highest parental education, N (%)** |  |  |  |  |  |
| Primary and lower secondary education | 20,460 (8.3) | 83,583 (3.1) |  | 3,807 (12.4) | 100,236 (3.4) |
| Upper secondary education | 147,837 (59.7) | 1,095,439 (40.4) |  | 19,270 (62.6) | 1,224,006 (41.9) |
| Post-secondary education | 77,679 (31.4) | 1,444,574 (53.3) |  | 7,484 (24.3) | 1,514,769 (51.8) |
| Postgraduate education | 1,216 (0.5) | 76,124 (2.8) |  | 154 (0.5) | 77,186 (2.6) |
| Missing data | 387 (0.2) | 9,166 (0.3) |  | 53 (0.2) | 9,500 (0.3) |
| **Parent’s immigration status, N (%)** |  |  |  |  |  |
| Born in Sweden | 181,218 (73.2) | 2,275,333 (84.0) |  | 23,763 (77.2) | 2,456,506 (84.0) |
| Immigrant | 66,299 (26.8) | 433,189 (16.0) |  | 7,000 (22.8) | 469,035 (16.0) |
| Missing data | 62 (0.03) | 364 (0.01) |  | 5 (0.02) | 156 (0.01) |
| **Any paternal psychiatric disorder, N (%)** | 69,609 (28.1) | 163,382 (6.0) |  | 8,328 (27.1) | 224,663 (7.7) |
| **Any maternal psychiatric disorder, N (%)** | 47,779 (19.3) | 241,291 (8.9) |  | 13,705 (44.5) | 275,365 (9.4) |
| **Paternal externalizing disorder, N (%)** | 46,168 (18.7) | 59,262 (2.2) |  | 5,805 (18.9) | 99,625 (3.4) |
| **Maternal externalizing disorder, N (%)** | 18,481 (7.5) | 47,568 (1.8) |  | 7,884 (25.6) | 58,165 (2.0) |
| **Paternal suicidal behavior, N (%)** | 23,191 (9.4) | 44,188 (1.6) |  | 2,769 (9.0) | 64,610 (2.2) |
| **Maternal suicidal behavior, N (%)** | 16,726 (6.8) | 62,712 (2.3) |  | 6,020 (19.6) | 73,418 (2.5) |
| **Father's incarceration, N (%)** | 107,798 (43.5) | 77,214 (2.9) |  | 9,754 (31.7) | 175,258 (6.0) |
| **Mother's incarceration, N (%)** | 5,172 (2.1) | 5,629 (0.2) |  | 4,025 (13.1) | 6,776 (0.2) |
| **Coresiding*, N (%)** |  |  |  |  |  |
| Parent coresiding with the child | 119,531 (51.1) | 2,056,233 (81.6) |  | 23,215 (78.5) | 2,567,271 (94.3) |
| Co-parent coresiding with the child | 213,826 (91.3) | 2,376,660 (94.4) |  | 12,671 (42.9) | 2,163,093 (79.4) |
| Missing data | 847 (0.4) | 10,125 (0.4) |  | 127 (0.4) | 10,845 (0.4) |
| *****Indicates whether the child had lived in the same area as his/her parent for at least 8 years of the first 10 years of life. Residential data were obtained from the DeSO (Demographic Statistical Areas) classification, based on which Sweden is divided into 5,984 geographical areas according to county and municipal boundaries, with data updated at the end of each year (Statistics Sweden, 2024). For coresiding information, individuals born in Sweden between 1982 and 2010 (N=2,752,991) were included. | | | | | |
| Information on parental violent convictions, parental incarceration, parental psychiatric disorders and parental suicidal behavior before the child’s 10^th^ birthday included. | | | | | |

| **Table** **S10.** Associations between parental violent convictions and offspring suicidal behavior, stratified by information on other parental factors. Reference group: offspring not exposed to paternal/maternal violent convictions. | | | | | | | | | | | | | |
| --- | --- | --- | --- | --- | --- | --- | --- | --- | --- | --- | --- | --- | --- |
|  |  | **Any suicidal behavior** | | |  | **Nonfatal self-harm** | | |  | **Suicide** | | | |
|  |  | **Paternal violent convictions** |  | **Maternal violent convictions** |  | **Paternal violent convictions** |  | **Maternal violent convictions** |  | **Paternal violent convictions** |  | **Maternal violent convictions** |  |
|  |  | HR (95% CI) |  | HR (95% CI) |  | HR (95% CI) |  | HR (95% CI) |  | HR (95% CI) |  | HR (95% CI) |  |
| Stratification by: | |  |  |  |  |  |  |  |  |  |  |  |  |
| **Parental psychiatric morbidity** | |  |  |  |  |  |  |  |  |  |  |  |  |
| Any diagnosis | | 1.68 (1.61–1.75) |  | 1.71 (1.60–1.84) |  | 1.68 (1.62–1.76) |  | 1.72 (1.60–1.84) |  | 1.63 (1.34–1.98) |  | 1.88 (1.36–2.59) |  |
| No psychiatric diagnosis | | 1.82 (1.78–1.87) |  | 2.28 (2.14–2.43) |  | 1.83 (1.79–1.87) |  | 2.27 (2.14–2.42) |  | 1.94 (1.74–2.17) |  | 3.32 (2.58–4.29) |  |
| **Parental externalizing disorders** | |  |  |  |  |  |  |  |  |  |  |  |  |
| Externalizing disorder | | 1.57 (1.49–1.66) |  | 1.42 (1.30–1.55) |  | 1.58 (1.49–1.67) |  | 1.42 (1.29–1.55) |  | 1.33 (1.04–1.70) |  | 1.64 (1.10–2.43) |  |
| No externalizing disorder | | 1.84 (1.81–1.89) |  | 2.26 (2.14–2.39) |  | 1.85 (1.81–1.89) |  | 2.26 (2.14–2.39) |  | 2.00 (1.80–2.21) |  | 3.05 (2.41–3.86) |  |
| **Parental suicidal behavior** | |  |  |  |  |  |  |  |  |  |  |  |  |
| Suicidal behavior | | 1.52 (1.42–1.62) |  | 1.53 (1.40–1.68) |  | 1.51 (1.42–1.62) |  | 1.53 (1.40–1.69) |  | 1.68 (1.25–2.24) |  | 1.79 (1.19–2.71) |  |
| No suicidal behavior | | 1.95 (1.91–1.99) |  | 2.35 (2.23–2.48) |  | 1.95 (1.91–1.99) |  | 2.35 (2.23–2.48) |  | 2.07 (1.88–2.29) |  | 3.28 (2.62–4.12) |  |
| **Parental incarceration** | |  |  |  |  |  |  |  |  |  |  |  |  |
| Incarceration | | 1.44 (1.39–1.50) |  | 1.44 (1.25–1.65) |  | 1.45 (1.40–1.51) |  | 1.43 (1.24–1.64) |  | 1.44 (1.20–1.73) |  | 1.60 (0.86–2.99) |  |
| No incarceration | | 1.73 (1.69–1.78) |  | 2.34 (2.23–2.46) |  | 1.73 (1.69–1.78) |  | 2.34 (2.22–2.46) |  | 1.79 (1.57–2.05) |  | 3.32 (2.67–4.12) |  |
| **Child–parent coresiding** | |  |  |  |  |  |  |  |  |  |  |  |  |
| Living in a different area | | 1.65 (1.60–1.69) |  | 2.01 (1.82–2.21) |  | 1.65 (1.60–1.69) |  | 2.01 (1.82–2.22) |  | 1.79 (1.57–2.04) |  | 2.44 (1.61–3.71) |  |
| Living in the same area | | 1.76 (1.71–1.82) |  | 2.35 (2.23–2.48) |  | 1.76 (1.71–1.82) |  | 2.35 (2.22–2.48) |  | 1.89 (1.62–2.20) |  | 3.27 (2.58–4.15) |  |
| Sample sizes for each stratum: **offspring of fathers** with violent convictions: paternal psychiatric disorder (N = 69,609), no paternal psychiatric disorder (N = 177,970); paternal externalizing disorder (N = 46,168), no paternal externalizing disorder (N = 201,411); paternal suicidal behavior (N = 23,191), no paternal suicidal behavior (N = 224,388); paternal incarceration (N = 107,798), no paternal incarceration (N = 139,781); living in the same area (N = 119,531), living in a different area (N = 133,760). **Offspring of mothers** with violent convictions: maternal psychiatric disorder (N = 13,705), no maternal psychiatric disorder (N = 17,063); maternal externalizing disorder (N = 7,884), no maternal externalizing disorder (N = 22,884); maternal suicidal behavior (N = 6,020), no maternal suicidal behavior (N = 24,748); maternal incarceration (N = 4,025), no maternal incarceration (N = 26,743); living in the same area (N = 23,215), living in a different area (N = 6,215).  All models are adjusted for child's and parent's birth years and parental immigration status. Missing information on parental immigration was treated as a separate category. HR=hazard ratio, CI=confidence interval | | | | | | | | | | | | | |

| **Table S11.** Multivariable Cox regression models for the associations of parental violent offending and other parental factors with offspring suicidal behavior. | | | | | | |
| --- | --- | --- | --- | --- | --- | --- |
|  |  | **Any suicidal behavior** |  | **Nonfatal**  **self-harm** |  | **Suicide** |
|  |  | HR (95% CI) |  | HR (95% CI) |  | HR (95% CI) |
| **Paternal factors** | |  |  |  |  |  |
| Violent convictions | | 1.38 (1.34–1.41) |  | 1.38 (1.35–1.41) |  | 1.41 (1.25–1.59) |
| Psychiatric disorder | | 1.26 (1.33–1.40) |  | 1.36 (1.32–1.39) |  | 1.51 (1.33–1.72) |
| Suicidal behavior | | 1.47 (1.41–1.52) |  | 1.46 (1.41–1.52) |  | 1.49 (1.25–1.78) |
| Incarceration |  | 1.30 (1.27–1.33) |  | 1.30 (1.26–1.33) |  | 1.36 (1.20–1.54) |
| Living in a different area than the  child (ref. living in the same area) | | 1.57 (1.55–1.60) |  | 1.58 (1.26–1.33) |  | 1.70 (1.57–1.85) |
| **Maternal factors** | |  |  |  |  |  |
| Violent convictions | | 1.63 (1.55–1.71) |  | 1.63 (1.55–1.72) |  | 2.13 (1.70–2.67) |
| Psychiatric disorder | | 1.59 (1.55–1.63) |  | 1.59 (1.55–1.63) |  | 1.77 (1.56–2.01) |
| Suicidal behavior | | 1.75 (1.70–1.81) |  | 1.75 (1.69–1.81) |  | 1.78 (1.51–2.10) |
| Incarceration |  | 1.50 (1.39–1.62) |  | 1.50 (1.39–1.62) |  | 1.31 (0.92–1.86) |
| Living in a different area than the  child (ref. living in the same area) | | 1.30 (1.25–1.34) |  | 1.29 (1.25–1.33) |  | 1.52 (1.30–1.79) |
| Adjusted for child's and parent's birth years and parental immigration status. Missing information on parental immigration was treated as a separate category. HR=hazard ratio, CI=confidence interval | | | | | | |

# **Figures S2-S3.** Kaplan-Meier curves estimating the cumulative incidence of suicidal behavior for offspring unexposed to paternal/maternal violent convictions and other parental factors vs. offspring exposed to paternal/maternal violent convictions, further divided into subgroups based on the number of other parental factors to which the child had been exposed (ranging from 0 to 4; including psychiatric morbidity, suicidal behavior, incarceration, and the parent living apart from the child for most of the child’s childhood).

**
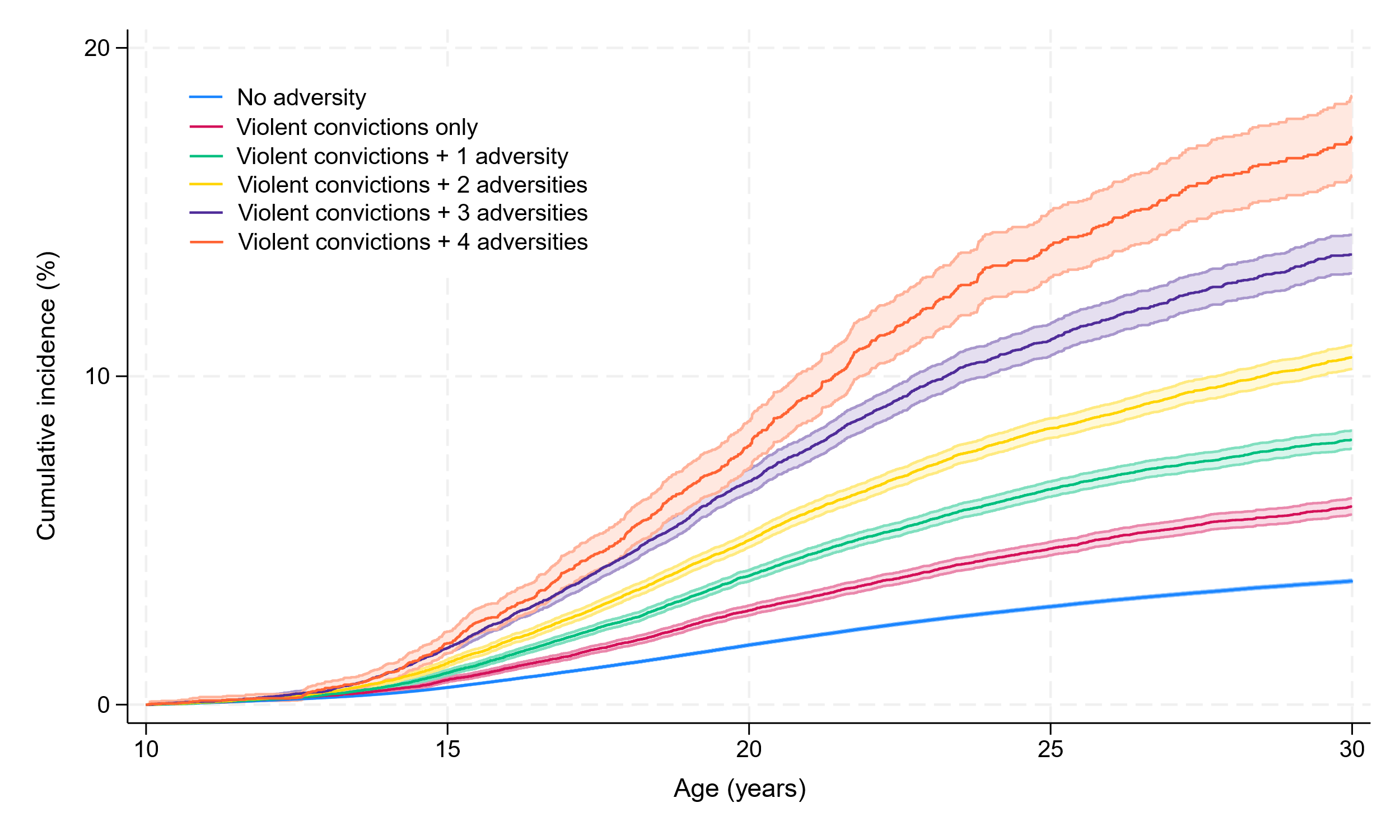
Figure S2.**

**Figure S3.**
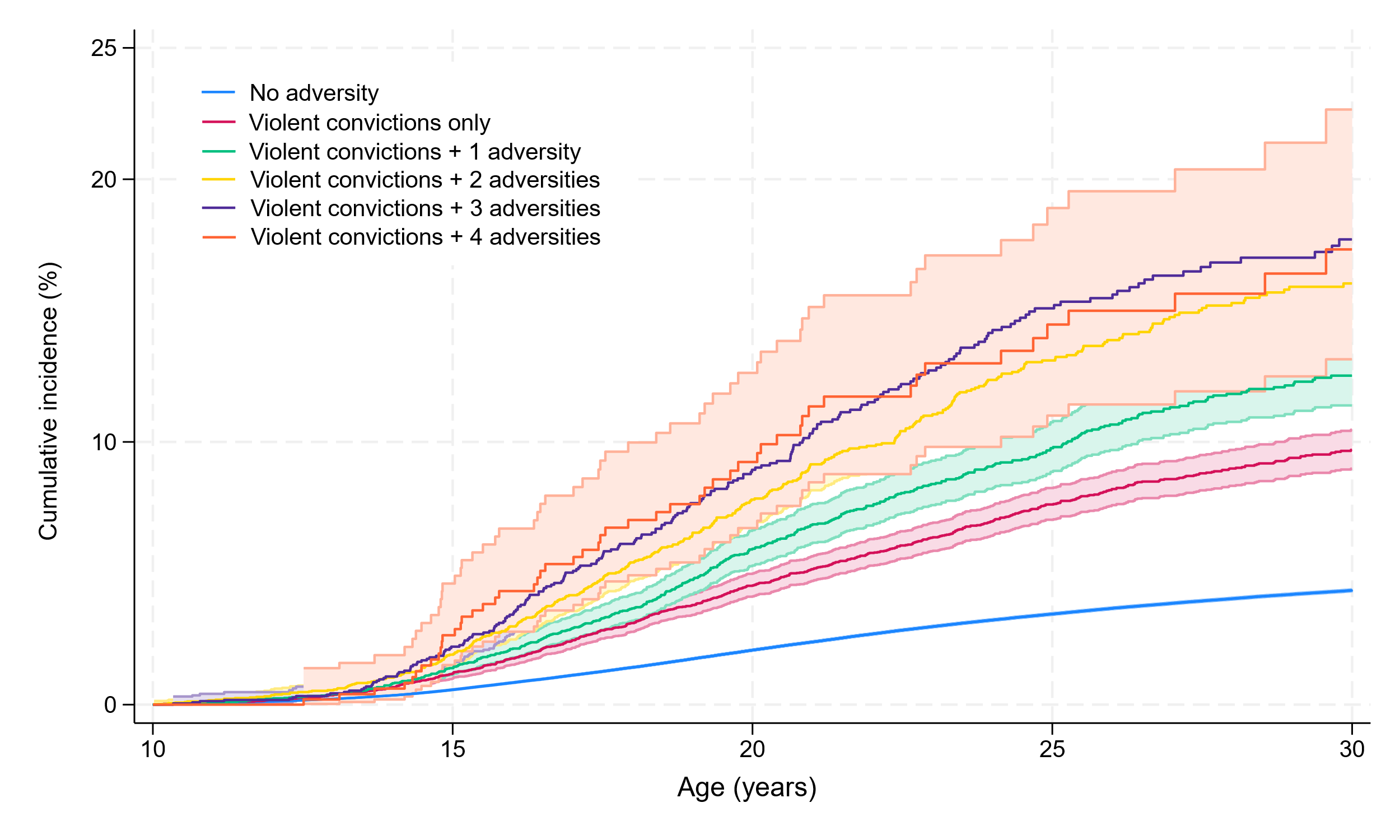


| **Table S12.** Results from Cox regression analyses for the association between paternal/maternal violent convictions and other adversities and offspring suicidal behavior. Exposure stratified by the number of parental factors to which the child had been exposed (ranging from 0 to 4, including psychiatric morbidity, suicidal behavior, incarceration, and the parent living apart from the child). Reference group: offspring not exposed to parental violent convictions or other parental adversity. | | | | |
| --- | --- | --- | --- | --- |
|  |  | **Offspring exposed to paternal adversity** |  | **Offspring exposed to maternal adversity** |
|  |  | HR (95% CI) |  | HR (95% CI) |
| Violent convictions only | | 1.58 (1.51–1.65) |  | 2.13 (1.97–2.29) |
| Violent convictions + 1 adversity | | 2.15 (2.07–2.22) |  | 2.70 (2.47–2.96) |
| Violent convictions + 2 adversities | | 2.80 (2.70–2.90) |  | 3.66 (3.31–4.04) |
| Violent convictions + 3 adversities | | 3.72 (3.56–3.89) |  | 4.12 (3.61–4.71) |
| Violent convictions + 4 adversities | | 4.65 (4.33–5.00) |  | 4.20 (3.21–5.50) |
| Models are adjusted for child's and parent's birth years and parental immigration status. Missing information on parental immigration was treated as a separate category.  Sample sizes for each stratum: offspring exposed to paternal violent convictions only (N = 68,292); to one additional adversity (N = 72,635), to 2 additional adversities (N = 55,549), to 3 additional adversities (N = 28,277), to 4 additional adversities (N = 8,538); offspring exposed to maternal violent convictions only (N = 12,498); to one additional adversity (N = 8,532), to 2 additional adversities (N = 5,306), to 3 additional adversities (N = 2,452), to 4 additional adversities (N = 642) HR=hazard ratio, CI=confidence interval | | | | |

| **Table S13.** Cumulative incidence (95% confidence interval) of suicidal behavior (with events and deaths of undetermined intent excluded) by age 30 among offspring with none, one, or two parents convicted of violent offenses. | | | | | | |
| --- | --- | --- | --- | --- | --- | --- |
|  |  | **Offspring of parents without violent convictions** (N = 2,689,895) |  | **Offspring with one parent convicted of violent offenses** (N = 254,793) |  | **Offspring with both parents convicted of violent offenses** (N = 11,777) |
|  |  | Cumulative incidence, % (95% CI) |  | Cumulative incidence, % (95% CI) |  | Cumulative incidence, % (95% CI) |
| **Female offspring** | |  |  |  |  |  |
| Suicidal behavior | | 3.2 (3.1–3.2) |  | 7.4 (7.3–7.6) |  | 14.3 (13.1–15.6) |
| Nonfatal intentional  self-harm | | 3.1 (3.1–3.2) |  | 7.4 (7.2–7.6) |  | 14.2 (12.9–15.5) |
| Suicide | | 0.1 (0.1–0.1) |  | 0.2 (0.2–0.3) |  | 0.5 (0.3–0.8) |
| **Male offspring** | |  |  |  |  |  |
| Suicidal behavior | | 1.9 (1.9–1.9) |  | 5.1 (4.9–5.3) |  | 10.5 (9.3–11.8) |
| Nonfatal intentional  self-harm | | 1.7 (1.7–1.8) |  | 4.8 (4.6–5.0) |  | 10.1 (9.0–11.4) |
| Suicide | | 0.2 (0.2–0.2) |  | 0.5 (0.4–0.5) |  | 0.7 (0.4–1.1) |

| **Table S14.** Results from Cox regression analyses for the population-level associations between parental violent convictions and offspring suicidal behavior, with events and deaths of undetermined intent excluded. | | | | |
| --- | --- | --- | --- | --- |
|  |  | **Male offspring** |  | **Female offspring** |
|  |  | HR (95% CI) |  | HR (95% CI) |
| **Any suicidal behavior** | |  |  |  |
| One parent with violent convictions | | 2.54 (2.45–2.64) |  | 2.22 (2.16–2.29) |
| Two parents with violent convictions | | 5.06 (4.50–5.69) |  | 4.24 (3.86–4.65) |
| **Nonfatal intentional self-harm** | |  |  |  |
| One parent with violent convictions | | 2.60 (2.50–2.70) |  | 2.23 (2.16–2.29) |
| Two parents with violent convictions | | 5.31 (4.71–5.97) |  | 4.24 (3.87–4.66) |
| **Suicide** |  |  |  |  |
| One parent with violent convictions | | 2.06 (1.82–2.34) |  | 1.98 (1.64–2.40) |
| Two parents with violent convictions | | 2.97 (1.84–4.80) |  | 4.84 (2.79–8.40) |
| Adjusted for child's and parent's birth years, and parental immigration status. Missing information on parental immigration was treated as a separate category.  HR=hazard ratio, CI=confidence interval | | | | |

| **Table S15.** Results from Cox regression analyses for the population-level associations between parental violent convictions and offspring suicidal behavior, with the sample restricted to children (N=2,140,955) whose parents were born from 1958 onwards. | | | | |
| --- | --- | --- | --- | --- |
|  |  | **Male offspring** |  | **Female offspring** |
|  |  | HR (95% CI) |  | HR (95% CI) |
| **Suicidal behavior** | |  |  |  |
| One parent with violent convictions | | 1.88 (1.82–1.95) |  | 1.91 (1.85–1.97) |
| Two parents with violent convictions | | 3.09 (2.75–3.48) |  | 3.38 (3.06–3.74) |
| Adjusted for child's and parent's birth years, and parental immigration status. Missing information on parental immigration was treated as a separate category. HR=hazard ratio, CI=confidence interval  HR=hazard ratio, CI=confidence interval   \| **Table S16.** Results from Cox regression analyses for the population-level associations between parental violent convictions and offspring suicidal behavior, with exposure including all parental violent convictions before the child's 30th birthday. \| \| \| \| \| \| --- \| --- \| --- \| --- \| --- \| \|  \|  \| **Male offspring** \|  \| **Female offspring** \| \|  \|  \| HR (95% CI) \|  \| HR (95% CI) \| \| **Suicidal behavior** \| \|  \|  \|  \| \| One parent with violent convictions \| \| 1.91 (1.86–1.96) \|  \| 2.02 (1.97–1.07) \| \| Two parents with violent convictions \| \| 3.29 (3.04–3.55) \|  \| 3.61 (3.38–3.87) \| \| Adjusted for child's and parent's birth years, and parental immigration status. Missing information on parental immigration was treated as a separate category.  HR=hazard ratio, CI=confidence interval \| \| \| \| \| | | | | |

# **References**

Brottsförebyggande rådet. (2024). *Swedish Crime Statistics*. Retrieved January 10, 2024 from <https://bra.se/bra-in-english/home.html>

Zagai, U., Lichtenstein, P., Pedersen, N. L., & Magnusson, P. K. E. (2019). The Swedish Twin Registry: Content and Management as a Research Infrastructure. *Twin Research and Human Genetics*, *22*(6), 672-680, Article Pii s1832427419000999. <https://doi.org/10.1017/thg.2019.99>
